# Microscopic basis for kinetic gating in cytochrome c oxidase: insights from QM/MM analysis†
†Electronic supplementary information (ESI) available: Benchmark results for the DFTB3 approach for the copper site and the doubly protonated glutamate are included; validation of DFTB3/MM interaction using several relevant models is also included. Additional PMF results obtained with a different variant of DFTB are included to demonstrate that the qualitative trends are robust. Comparison of PMF and microscopic p*K*_a_ calculations is included as further validation. Other materials include the isomerization of the cis/trans conformers of the Glu286 side chain, proton transfer PMF from Asp132 to the “serine zone”, and additional snapshots from various simulations. See DOI: 10.1039/c4sc01674b



**DOI:** 10.1039/c4sc01674b

**Published:** 2014-10-06

**Authors:** Puja Goyal, Shuo Yang, Qiang Cui

**Affiliations:** a Department of Chemistry and Theoretical Chemistry Institute , University of Wisconsin–Madison , 1101 University Avenue , Madison , WI 53706 , USA . Email: cui@chem.wisc.edu

## Abstract

Understanding the mechanism of vectorial proton pumping in biomolecules requires establishing the microscopic basis for the regulation of both thermodynamic and kinetic features of the relevant proton transfer steps. For the proton pump cytochrome c oxidase, while the regulation of thermodynamic driving force for key proton transfers has been discussed in great detail, the microscopic basis for the control of proton transfer kinetics has been poorly understood. Here we carry out extensive QM/MM free energy simulations to probe the kinetics of relevant proton transfer steps and analyze the effects of local structure and hydration level. We show that protonation of the proton loading site (PLS, taken to be a propionate of heme a_3_) requires a concerted process in which a key glutamic acid (Glu286H) delivers the proton to the PLS while being reprotonated by an excess proton coming from the D-channel. The concerted nature of the mechanism is a crucial feature that enables the loading of the PLS before the cavity containing Glu286 is better hydrated to lower its p*K*_a_ to experimentally measured range; the charged rather than dipolar nature of the process also ensures a tight coupling with heme a reduction, as emphasized by Siegbahn and Blomberg. In addition, we find that rotational flexibility of the PLS allows its protonation before that of the binuclear center (the site where oxygen gets reduced to water). Together with our recent study (P. Goyal, *et al.*, *Proc. Natl. Acad. Sci. U. S. A.*, 2013, **110**, 18886–18891) that focused on the modulation of Glu286 p*K*_a_, the current work suggests a mechanism that builds in a natural sequence for the protonation of the PLS prior to that of the binuclear center. This provides microscopic support to the kinetic constraints revealed by kinetic network analysis as essential elements that ensure an efficient vectorial proton transport in cytochrome c oxidase.

## Abstract 2

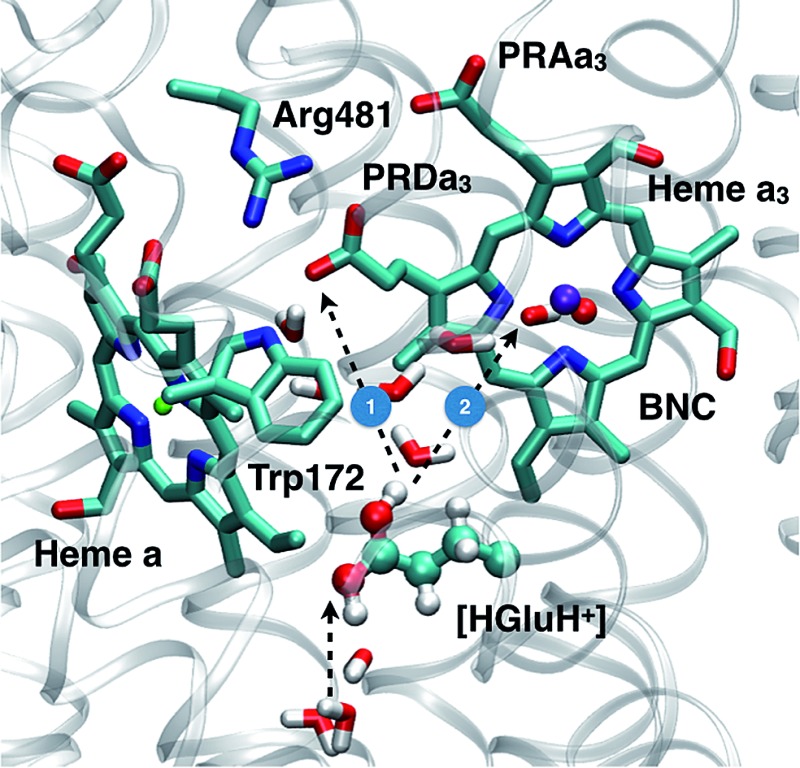
Understanding the mechanism of vectorial proton pumping in biomolecules requires establishing the microscopic basis for the regulation of both thermodynamic and kinetic features of the relevant proton transfer steps.

## Introduction

Proton pumping is an essential process in bioenergetics.^1^ For example, impairment of proton pumping function in mitochondria has been implicated in several serious human diseases.^2–6^ There is also considerable interest in developing artificial (bio)systems for pumping protons for various energy related applications.^7,8^ Therefore, understanding the microscopic mechanism that ensures the vectorial nature of proton pumping is of fundamental, biomedical and practical significance. Along this line, although much is known for the simpler light-activated proton pumps such as bacteriorhodopsin,^9^ the mechanism for the more complex multi-subunit proton pumps remains poorly understood.

**Fig. 1:**
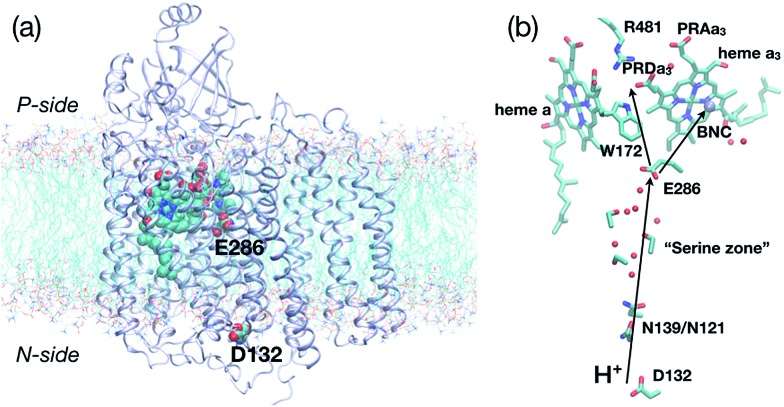
Key residues and co-factors that mediate proton transfers in cytochrome c oxidase. (a) A full protein model (based on the crystal structure,^36^; 1M56, for *Rhodobacter sphaeroides* CcO) embedded in a lipid bilayer to illustrate the approximate positions of the “proton antenna”, D132, the key glutamate, E286, and the heme groups. The non-hydrogen atoms in these groups are shown in van der Waals representation, and the rest of the protein in ribbon. (b) Key residues near the hydrophobic cavity (the region surrounding E286 and delimited by PRDa_3_ at the top), the D-channel (the water-lined “channel” between D132 and E286) and general proton pathways to and from E286. The propionate D of heme a_3_ (PRDa_3_) is taken as the proton loading site (PLS) in this study, although proton transfer between PRDa_3_ and the propionate A of heme a_3_ (PRAa_3_) is also studied.

A case in point is cytochrome c oxidase (CcO)^10–14^ (Fig. 1a), which is a highly efficient *trans*-membrane proton pump present in bacterial and inner mitochondrial membranes. It catalyzes the exothermic reduction of molecular oxygen to water and harnesses the energy released thereby to carry out vectorial proton transfer across the membrane against a proton concentration gradient. Thanks to extensive experimental^10–16^ and computational^17–33^ studies, much is known about the structure of CcO^34–38^ for several redox states and the kinetics of key electron/proton transfer steps. Despite these impressive progress, the fundamental question remains: what prevents the back flow of proton(s) from the P-side to the N-side of the membrane through CcO, following the proton concentration gradient? Different proposals have been suggested in the literature that included side chain isomerization of Glu286,^21,27,39^ orientation of water wires in the active site region,^26^ and free energy penalty associated with proton transfer through a hydrophobic cavity.^18^ Our recent atomistic simulations,^32^ however, suggested that Glu286 isomerization and water wire orientation alone are unlikely robust gating elements in CcO, highlighting the importance of explicitly considering proton transfer kinetics in the discussion of gating.^24,40^

A particularly interesting and elegant study in this context is that of Kim and Hummer,^28,41^ who constructed a set of minimal kinetic models for coupled electron/proton transfers in CcO based on chemical master equations.^42^ These models allowed them to identify patterns in the electron/proton transfer rate constants that would lead to efficient forward proton pumping and minimal proton back flow fluxes. Two sets of “kinetic gating constraints” for ensuring efficient pumping emerged from their analysis:^28^ (1) proton transfer to the proton loading site (PLS) is strongly coupled to the reduction of a nearby co-factor (*e.g.*, heme a); (2) proton transfer to the PLS precedes the proton transfer to the binuclear center (BNC, see Fig. 1b), and loading of the PLS enhances the recombination of electron and proton at the BNC.

Although these observations make intuitive sense from a functional consideration, constructing microscopic models that are consistent with these constraints has not been straightforward. The original work suggested water wire reorientation coupled to heme a reduction as one possible model for the control of proton transfer destination and kinetics.^26,43^ Since the model was motivated by MD simulations without including an excess proton in the region,^26^ the relevance should be re-evaluated with microscopic simulations that explicitly study proton transfers. A number of computational studies have examined proton transfers in CcO using various approaches;^17,18,20,22,23,44,45^ although insights were gained, the differences and limitations in the computational models led to the lack of consensus (for more discussions, see ESI†). For example, the minimum energy path (MEP) analysis by Siegbahn and Blomberg^22,23^ using DFT and cluster models pointed to a concerted proton transfer mechanism; the charged rather than dipolar nature of the transition state was suggested to be essential to the coupling between protonation of the PLS and heme a reduction. Although insightful, the study didn't include thermal fluctuations of the protein, which was known to be essential to reactions in enzymes,^46–48^ especially for the transport of charged species.^49–53^ Indeed, the concerted mechanism was not considered in most experimental or computational studies; for example, the analyses of Warshel and coworkers also raised the possibility of the concerted mechanism,^17^ which appears to be abandoned in the later study^18^ but then brought back to discussion in the latest work.^19^ Clearly, it is essential to (re)examine the microscopic mechanism of proton transfers in CcO with all the relevant groups, their thermal fluctuations and the complete enzyme environment included explicitly; this is the focus of this work.

A specific motivation for this study is our recent work that probed the thermodynamic driving force for proton transfers in CcO. Using both microscopic (hybrid QM/MM simulations with thermodynamic integration^54^) and macroscopic models (Poisson–Boltzmann with Linear Response^55–57^ and Multi-Conformer-Continuum Electrostatics^58^), we found that, when the PLS (assumed to be PRDa_3_, see below) is unloaded, the pK′7 of the key residue, Glu286, is very high and therefore it is unlikely to give up its proton to any site; the main reason is that the area surrounding Glu286 is hydrophobic in nature (see Fig. 1b) and therefore there is a large desolvation penalty for Glu286 ionization. Once the PLS is loaded, largely independent of the protonation state of Glu286, the cavity between Glu286 and PRDa_3_ expands^33^ due to the weakening of hydrogen bonding interactions associated with a charge neutral PRDa_3_, allowing the local hydration level to increase substantially. The enhancement of the hydration level and removal of the negative charge from PRDa_3_ work synergistically to lower the pK′7 of Glu286 by a significant amount, making possible for it to donate a proton to the BNC. Thus, this mechanism naturally suggests that loading of the PLS precedes and facilitates proton transfer to the BNC. A key issue not resolved, however, is the molecular mechanism that loads the PLS, which we address in this work. Specifically, we report QM/MM free energy (potential of mean force, PMF) calculations for several relevant proton transfer pathways in different redox/titration states of CcO. The results provide microscopic support to the kinetic gating phenomena discussed for proton pumping in CcO.^24,28,40,41^ Some of the key features of our mechanism (the importance of a concerted proton transfer and its tight coupling to heme a reduction) also qualitatively support the pioneering analysis of Siegbahn and Blomberg based on B3LYP calculations of cluster models (with ∼200 atoms) of CcO.^22,23^

Below, we first summarize the computational models and methods involved. Next, we present free energy results related to the key proton transfer steps in CcO, together with their dependence on protein structure and cavity hydration level. This is followed by discussions on the validity of different proton transfer mechanisms studied and their connection to experimental studies (connections to previous computational studies are drawn in the ESI†). Finally, we conclude with a summary of key insights drawn from this study and scope for continuing future work.

## Computational methods and enzyme models

### Basic simulation setup and strategies

Details of the enzyme model and simulation protocols are described in our earlier work.^31–33^ Briefly, we study several states of the enzyme relevant to the **P**_R_ → **F** transition, which has been analyzed extensively experimentally. As in ref. 33, the states are also denoted with a six-letter notation, such as PDD-ROg, where the first three letters indicate the protonation states (protonated or deprotonated) of Glu286, PRDa_3_ and the oxygenous ligand of Cu_B_, the next two letters indicate the oxidation states (oxidized or reduced) of heme a and heme a_3_, while the last letter indicates the force field used for the co-factors (“*g*” for the Ghosh-set^31^ and “*j*” for the Johansson-set^59^); the oxidation and protonation states of other key groups in the enzyme are summarized in Table 1. To simplify discussions, we also refer to PDD-ROg (before protonation of either the PLS or the BNC, with Glu286H) as **P**_R_, DPD-ROg (after “direct” protonation of the PLS, resulting in Glu286^–^) as **P**′R, PPD-ROg (after “concerted” protonation of the PLS, resulting in Glu286H) as **P**′′R and DPP-ORg (after both physical and chemical proton transfers and the electron transfer have been completed and Glu286 is deprotonated) as ′**F**. However, it should be noted that in ref. 33, **P**_R_, **P**′R, **P**′′R and ′**F** corresponded to PDD-OOj, DPD-OOj, PPD-OOj and DPP-OOj, respectively, which are consistent with the state assignments used in the experimental literature^12–14^ (see footnote of Table 1); we chose the charge states in the two sets of models such that the total charges of the active site are identical, thus it is meaningful to compare the results for p*K*_a_ (ref. 33) and proton transfers. In any case, both usages of **P**_R_, **P**′R, **P**′′R and ′**F** correspond to the same protonation states of Glu286, PRDa_3_ and the BNC and hence aid our discussion.

**Table 1:** Summary of different simulation setups used for the QM/MM proton transfer studies in this work

Input^*a*^	State^*b*^	Redox/titration patterns^*c*^	Proton transfer	Parameters^*d*^	Cavity
1M56	PDD-RO	E286H; PRDa_3_^–^; Cu_B_^2+^–OH^–^; Fe_a_(ii); Tyr288H	E286H → PRDa_3_	*g*	Small
1M56	PDD-OO	E286H; PRDa_3_^–^; Cu_B_^2+^–OH^–^; Fe_a_(iii); Tyr288H	E286H → PRDa_3_	*g*	Small
1M56+9w	PDD-RO	E286H; PRDa_3_^–^; Cu_B_^2+^–OH^–^; Fe_a_(ii); Tyr288H	E286H → PRDa_3_	*g*	Small
pre**P**′′R	PDD-RO	E286H; PRDa_3_^–^; Cu_B_^2+^–OH^–^; Fe_a_(ii); Tyr288H	E286H → PRDa_3_	*j*,*g*	Small
′**F**	PDD-RO	E286H; PRDa_3_^–^; Cu_B_^2+^–OH^–^; Fe_a_(ii); Tyr288H	E286H → PRDa_3_	*j*,*g*	Large
1M56	PDD-RO	E286H; PRDa_3_^–^; Cu_B_^2+^–OH^–^; Fe_a_(ii); Tyr288H	H_3_O^+^ → PRDa_3_	*g*	Small
1M56	PDD-OO	E286H; PRDa_3_^–^; Cu_B_^2+^–OH^–^; Fe_a_(iii); Tyr288H	H_3_O^+^ → PRDa_3_	*g*	Small
′**F**	PDD-RO	E286H; PRDa_3_^–^; Cu_B_^2+^–OH^–^; Fe_a_(ii); Tyr288H	H_3_O^+^ → PRDa_3_	*j*,*g*	Large
pre**P**′′R	PPD-RO	E286H; PRDa_3_H; Cu_B_^2+^–OH^–^; Fe_a_(ii); Tyr288H	PRDa_3_H → Cu_B_^2+^–OH^–^	*j*,*g*	Small
′**F**	PPD-RO	E286H; PRDa_3_H; Cu_B_^2+^–OH^–^; Fe_a_(ii); Tyr288H	PRDa_3_H → PRAa_3_	*j*,*g*	Large
1M56	PDD-RO	E286H; PRDa_3_^–^; Cu_B_^2+^–OH^–^; Fe_a_(ii); Tyr288H	D132H → H3O^+^	*g*	Small

^*a*^All QM/MM calculations use the GSBP (Generalized Solvent Boundary Potential) approach, although some of them use a snapshot from a PBC simulation as the starting structure. Input structure: 1M56: starting coordinates taken from the crystal structure, with GCMC addition of water molecules;^31^ 1M56+9w: 6 additional water molecules added to 1M56 structure in the region near Glu286 and 3 near PRDa_3_; ′**F**: local GSBP simulation starting coordinates taken from a snapshot of the ′**F**-state PBC simulation. pre**P**′′R: local GSBP simulation starting coordinates taken from a snapshot of the pre-**P**′′R state PBC simulation (which features E286H, PRDa_3_^–^, Cu_B_^2+^–OH^–^ and a hydronium in the hydrophobic cavity).

^*b*^The states (prior to the proton transfer) are labeled with a 5 character notation. The first three letters indicate the protonation state (Protonated or Deprotonated) of Glu286, propionate D of heme a_3_ (PRDa_3_), the ligand of Cu_B_ (hydroxide (D) or water (P)). The last two letters indicate the reduction state (Reduced or Oxidized) of heme a and Cu_B_, respectively.

^*c*^Other co-factors are fixed as: Cu_A_ oxidized, Fe_a3_(iv)=O^2–^, His334H.

^*d*^Parameters for the metal co-factors: “*j*” uses the Johansson set^59^ and “*g*” uses the Ghosh set.^31^ The Ghosh parameters have a neutral Tyr288 and the Johansson parameters have a deprotonated, anionic Tyr288. Therefore, the net charge of hemes a and a_3_, Cu_B_ and Tyr288 in the **P**_R_ (PDD-OO) state with the Johansson parameters is identical to that of the PDD-RO state with the Ghosh parameters. In the latter, the extra electron resides on heme a. Notation “*j*,*g*” means that the Johansson set is used in the PBC simulations, and the Ghosh set used in the subsequent QM/MM-GSBP simulations.

Because of the considerable computational expense associated with QM/MM calculations using periodic boundary conditions (PBC) for a large membrane protein like CcO, our approach is to use the Generalized Solvent Boundary Potential (GSBP) protocol in a DFTB/MM framework.^50,60,61^ Since the GSBP approach treats the parts of the protein distant from the region of interest as fixed (although the mobile region in our GSBP simulations still contains ∼8000 atoms with the dimensions of this orthorhombic inner region centered at Glu286 being 40 Å × 38 Å × 56 Å; for simulations involving D132 in the proton transfer, the inner region is extended an additional 12 Å “below” D132), it is important to understand the limitations in the conformational response, if any, to changes in titration states of different groups involved in the proton transfers. To this end, as reported in ref. 33, we have performed comparisons of conformational flexibility and solvation changes of the active site region between PBC and GSBP simulations for a number of states that differ in the protonation states of Glu286, PRDa_3_ and the BNC. These analyses led to the conclusion that although flexibility of the loop that bears Trp172 and hydration changes of the hydrophobic cavity around Glu286 are underestimated by GSBP simulations, if a representative snapshot from PBC simulations for a particular enzyme state is used as the input structure for building a GSBP model, subsequent GSBP simulations recover all the key properties of the corresponding PBC simulations. In fact, this is a useful strategy to combine extensive MM PBC simulations with QM/MM-GSBP calculations for probing effects due to changes in protein structure and/or local solvation on chemical reactions in the active site.^62^

**Fig. 2:**
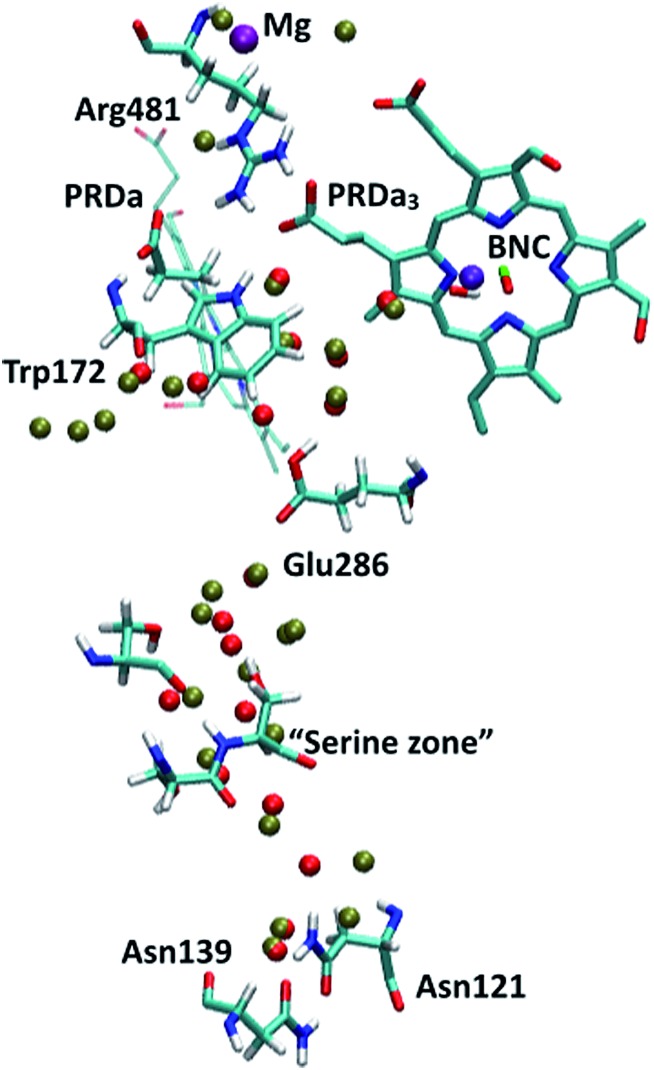
**P_R_**-state snapshot for the ; 1M56 model (colored by atom type). The tan-colored spheres represent water oxygen atoms in the ; 1M56+9w model (note the extra water molecules in the D-channel, near Trp172 and near PRDa_3_/Mg compared to ; 1M56). Note that in the snapshots in this and following figures, most protein atoms are not shown for clarity although they are included in the calculations (see Methods).

**Fig. 3:**
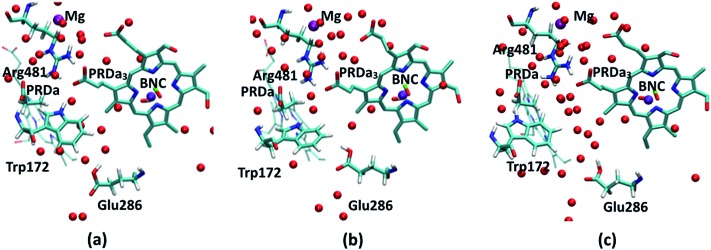
**P_R_**-state snapshots for the (a) ; 1M56 (b) pre**P′′R** (c) **′F** models. The red spheres represent water oxygen atoms. For discussion of orientation of the acidic proton in Glu286H, see ESI.†

Regarding the GSBP set-up (summarized in Table 1), two models (; 1M56 and ; 1M56+9w) are based on the crystal structure^36^ (PDB code ; 1M56); the number and location of water molecules in the hydrophobic cavity were determined based on Grand Canonical Monte Carlo (GCMC) simulations,^31,63^ with ; 1M56+9w having 6 extra water molecules in the D-channel or near Trp172 and 3 extra near PRDa_3_/Mg compared to ; 1M56, as illustrated in Fig. 2 (to overcome potential convergence issues in conventional GCMC, 8 water molecules were initially placed in “vacancies” in the D-channel in the ; 1M56 model, of which GCMC deleted 2; besides, GCMC added 3 extra water molecules near PRDa_3_/Mg).^33^ Another model, ′**F**, is based on a snapshot from a PBC simulation of the ′**F** state, in which the hydrophobic cavity has a substantially enhanced level of hydration (compare Fig. 3a and c).^33^ Finally, we carry out simulations in an additional GSBP setup referred to as pre**P**′′R (see Fig. 3b), which is based on a snapshot from a PBC simulation with Glu286 protonated and a hydronium in the hydrophobic cavity, corresponding to a configuration right before the formation of the **P**′′R state. In this simulation (see Fig. 6a), “downward” rotation of PRDa_3_ causes weakening of the salt-bridge interaction between Arg481-PRDa_3_ as well as a slight displacement of Trp172; however, the cavity hydration level is comparable to that in the **P**_R_ state (consistent with the fact that PRDa_3_ has not yet been protonated), implying a possible proton transfer pathway to either PRDa_3_ or the BNC *via* the few water molecules that the cavity holds. Therefore, by imposing the same oxidation/protonation states of the key groups but using different initial structures in the various QM/MM-GSBP simulations, we will be able to gain useful insights into the importance of factors such as local hydration level and side chain conformation on the proton transfer kinetics. Note that throughout this work, “downward/upward” orientation of a residue implies an orientation in which the side-chain points towards the negative (N)/positive (P) side of the membrane (see Fig. 1).

### QM/MM set-up

All proton transfer studies are carried out in a QM/MM framework using DFTB as the QM method;^50,64–66^ the EXGR link atom scheme^67^ is adopted for the QM/MM boundary except in cases where the link atom is placed between the C_α_ and C_β_ atoms of a residue which necessitates use of the DIV scheme.^67,68^ The QM region typically includes the proton donor group, acceptor group and intervening water molecules, thus slightly different QM regions are used for studying different proton transfer processes. The BNC is treated as MM in most studies except for the proton transfer between PRDa_3_ and Cu_B_-bound OH^–^ in the pre**P**′′R model, for which Cu_B_ and its ligands as well the side chain of Tyr288 are also included in the QM region. The size of the QM region thus ranges from 30 to 78 atoms in different QM/MM calculations. In all the snapshots from the PMF simulations, the QM region atoms are shown in the CPK representation.

The specific DFTB variant used for most PMF calculations is DFTB3^69^ with fitted Hubbard charge derivatives^70^ in combination with the ‘MIO’ parameter set and addition of a Gaussian term to the O–H repulsive potential in the 1.1–1.6 Å distance range.^50,71^ We refer to this combination as DFTB3/MIO/fit+gaus. In ESI,† we also show results from some PMF calculations and QM/MM-TI-based p*K*_a_ calculations^72^ carried out with the DFTB3-diag/MIO+gaus variant using parameter set 5 in Table 2 of ref. 70 (*i.e.*, the same DFTB3-diag+gaus variant discussed in ref. 71). We note that the DFTB3-diag/MIO+gaus variant has ∼7 kcal mol^–1^ error in the relative proton affinities of a carboxylic acid (the analog of Glu/Asp side chains and propionic acid of heme) and small water clusters, thus reducing the quantitative accuracy of the free energy profiles. By contrast, the DFTB3/MIO/fit+gaus variant features a much lower error of ∼2 kcal mol^–1^ for this quantity. The qualitative trends in the results, however, are consistent between the two sets of DFTB calculations, further supporting the findings of these calculations. We also note that several recent articles^73–75^ discussed limitations of the DFTB3 model in treating bulk water and hydration of proton/hydroxide in condensed phase. We openly acknowledge these limitations^71^ and regard systematically improving DFTB3 for treating water in different environments as one of the essential topics for our continuing DFTB developments. However, we emphasize that the proton transfer barriers are not severely affected by these limitations; our studies^71,76^ never encountered errors of more than 1–2 kcal mol^–1^ due to over-solvation of the proton. As discussed below, the different pathways we aim to distinguish involve much larger differences in barriers and therefore the qualitative trends are robust.

Simulations using the pre**P**′′R model are carried out using the ‘3OB’ parameter set^77^ (we refer to this variant as DFTB3/3OB) because of the compatibility of this parameter set with the Cu parameters recently developed in our group.^78^ This variant has ∼5 kcal mol^–1^ error in the relative proton affinities of a carboxylic acid and small water clusters. As shown in ESI,† the method well describes the proton affinities of two copper complexes (with and without the cross-linked Tyr) modeled after the BNC. Performance of the Cu parameters for condensed phase simulations has also been tested by reduction potential calculations for the blue-copper proteins, plastocyanin and rusticyanin, with the results showing that these parameters can describe structural and energetic properties well.^78^

For the MM part, the protein is described with the CHARMM22 force field^79^ (including CMAP^80^) and water treated with modified TIP3P.^81^ As shown in ESI† and ref. 76, DFTB3/MM interactions work adequately as compared to full QM (DFTB3, B3LYP or MP2) calculations or available solvation free energies of small solutes. We also test the potential importance of including electronic polarization for groups near the region of interest by adopting a simple charge-scaling scheme for selected residues.^82,83^ As discussed in ref. 33, charge-scaling and using different force field parameters for the BNC were found to have a rather minor impact on the computed pK′7 of Glu286 and general behavior of the active site region.

### Proton transfer potentials of mean force (PMF)

Although the cluster-MEP studies of Siegbahn and Blomberg^22,23^ have been insightful, quite a number of studies^46–48,50,51,84^ emphasized the importance of including thermal fluctuations, especially for processes that involve transport of charged species. Therefore, it is essential to carry out PMF simulations in the enzyme and compare to the MEP analyses of cluster models. Throughout this work, we assume that PRDa_3_ is at least a transient proton loading site, which is also assumed in most computational studies of proton transfers in CcO^18,21,23,85,86^ given the unique location of PRDa_3_ (see Fig. 1b); nevertheless, we also consider the possibility of proton transfer from PRDa_3_ to PRAa_3_. The PMF calculations are carried out using the standard umbrella sampling technique^87^ in combination with the weighted histogram analysis method (WHAM).^88,89^ The number of windows in the various PMFs ranges from 9 to 35 with force constants ranging from 70 to 1000 kcal mol^–1^ (the *ζ* coordinate is dimensionless; windows are typically placed at intervals of 0.1 along *ζ*). The typical production sampling per window is 450–600 ps (except the N139S/N121S simulations in which the production sampling per window is ∼1.4 ns). The total production data per window is divided into 3–4 blocks of 100–200 ps in order to obtain an estimate of the average PMF and the associated error bar (a 90% confidence interval of the mean is chosen).

The reaction coordinate, denoted as *ζ* in the PMF results below, is based on the modified center of excess charge (mCEC) as described in ref. 90. The specific form of *ζ* used is,
1

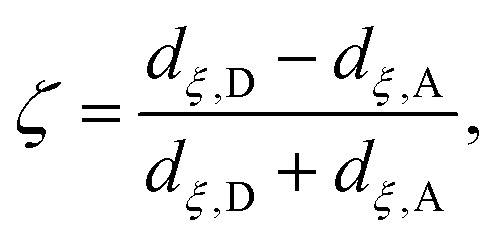

where *ξ* is the mCEC, D indicates the donor heavy atom and A denotes the acceptor heavy atom, and *d* indicates distance. Hence a *ζ* value of –1.0 represents a protonated donor while a value of +1.0 represents the excess proton being localized on the acceptor. For the “concerted” proton transfer pathway simulations (see below), which implicates a protonated Glu286 to relay proton transfer, the term 
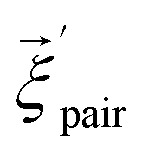
 (eqn (8) in ref. 90) is added to the mCEC definition to describe coupled protonation and deprotonation of the two carboxylate O atoms of Glu286. Our previous studies indicate that the combination of mCEC and *ζ* is able to describe complex proton transfer pathways,^51,90,91^ although those implicated in this study do not deviate significantly from linearity.

## Results and discussion

Our calculations focus on various proton transfer steps relevant to the **P**_R_ → **F** transition (the states and calculations are summarized in Table 1), although it is commonly assumed that the basic pumping mechanism is the same for the four sub steps of the functional cycle (*i.e.*, consumption of one oxygen molecule). As mentioned above in the Method section, by comparing results from different models (see Fig. 3), we are able to gain insights into the impact of cavity hydration on proton transfer and set bounds on the proton transfer barriers and thermodynamics.

### Proton transfer from Glu286H to PRDa_3_ is energetically unfavorable

Most previous studies assume that loading of the PLS, commonly taken to be PRDa_3_, occurs with a proton transfer from the charge-neutral Glu286H through one or a few intervening water molecules; an exception is the MEP analysis of cluster models by Siegbahn and Blomberg,^22,23^ who suggested that this proton transfer is energetically unfavorable, even after manually adding a few water molecules to better solvate Glu286. Except for the work of Warshel and co-workers,^17,18^ however, the free energy profile for this step has not been carefully studied. Therefore, we first analyze this process, which corresponds to the **P**_R_ → **P**′R transition in our notation. For this proton transfer to be a realistic mechanism for the loading of the PLS, the upper bound to the barrier needs to be ∼12 kcal mol^–1^, which corresponds to the measured time scale of ∼150 μs prior to the protonation of the BNC.^13^

### Proton transfer with a low level of cavity hydration

We first study the proton transfer from Glu286H to PRDa_3_ in models with a relatively low level of cavity hydration, ; 1M56 and pre**P**′′R (see Fig. 3a and b), to probe the effect of several factors that include: (i) the number of water molecules in the hydrophobic cavity, (ii) the oxidation state of heme a, and (iii) electronic polarization of nearby residues.

**Fig. 4:**
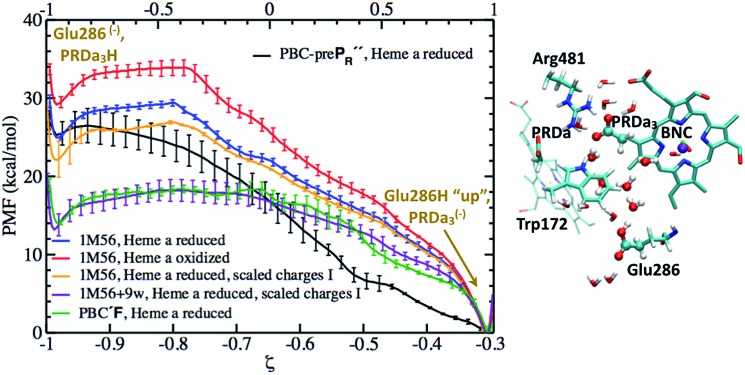
Computed PMFs for the proton transfer from Glu286H to PRDa_3_ using different enzyme models that differ in the level of cavity hydration, redox state of heme a and partial charges for nearby MM atoms; see Table 1 for the notation of different models. The lower *X* axis corresponds to the pre**P′′R** PMF, which goes only up to *ζ* ∼ –0.3 rather than 1.0 because the more static side-chain oxygen atom of Ser200 is used to define the mCEC, rather than one of the oxygen atoms of Glu286 due to the rotation of the Glu side chain during the proton transfer reaction; the top *X* axis corresponds to the PMFs for all other models. On the right, a snapshot is shown to illustrate the ; 1M56 model prior to the proton transfer; for additional snapshots from the PMF simulations, see Fig. S10.† Also see Fig. 3 for illustration of the hydration level in the different models.

Fig. 4 shows that with heme a reduced, both models predict the proton transfer from Glu286H to PRDa_3_^(–)^ to be highly unfavorable with similar endothermicities. The pre**P**′′R model shows the slight stability of a configuration in which Glu286H has undergone a large “upward” rotation to form a proton transfer pathway to PRDa_3_^(–)^ with a shorter water wire (see Fig. S10c and d†). This large rotation, however, is found to be unfavorable by almost ∼4 kcal mol^–1^. Thus the PMFs for these two models indicate that as long as the level of solvation of Glu286 is low, the number of water molecules mediating the proton transfer or the rotation of PRDa_3_/Glu286 do not play a significant role.

The effect of heme a oxidation is found to be ∼4 kcal mol^–1^, with heme a reduction favoring the proton transfer towards PRDa_3_^(–)^; this is consistent with the observation that heme a is spatially closer to PRDa_3_ than to Glu286. However, even the reduction of heme a is not able to prevent an easy and highly favorable backflow of the loaded proton (Fig. 4).

The unfavorable proton transfer from Glu286H to PRDa_3_^(–)^ is consistent with the high pK′7 computed for Glu286 using models that feature a low level of hydration in the cavity.^19,33^ However, another contributing factor is that, prior to proton transfer, PRDa_3_^(–)^ forms a favorable salt-bridge with Arg481, whose strength might be overestimated with a non-polarizable MM model.^82,83^ For a relatively simplified model to consider electronic polarization, Stuchebrukhov *et al.* ^82,83^ proposed to scale the partial charges of charged residues buried in the protein interior by 
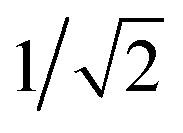
; the scaling factor was motivated by the typical value of high-frequency dielectric constant, although recent comparison of computed and experimental binding free energies of charged ligands in proteins pointed to much more modest empirical scaling factors.^92^ In our calculations, since PRDa_3_ is in the QM region, it is meaningful to only scale the charges of surrounding charged residues. As shown in Fig. 4, scaling the charges of only Arg481 (scaled charges I) leads to the protonation of PRDa_3_ being more favorable by a modest value of ∼4 kcal mol^–1^. Fig. S11† shows that additionally scaling the charges of other nearby charged groups, *viz.* Arg482, PRAa_3_, PRDa, PRAa while including/excluding Cu_B_ with its ligands, leads to even lesser change in the ability of PRDa_3_ to accept a proton. The PMF for proton transfer in the region close to Glu286 does not change in the different charge-scaling schemes, consistent with the fact that there are no charged groups very close to Glu286, as also highlighted in ref. 33.

In short, the different PMFs indicate that, as long as the hydration level of the cavity remains low, proton transfer from Glu286H → PRDa_3_^(–)^ has a barrier of at least ∼22–24 kcal mol^–1^ with the endothermicity being at least ∼20 kcal mol^–1^. Thus it is important to study the proton transfer in question with a model that features a better hydrated cavity.

### Proton transfer with a high level of cavity hydration

In the QM/MM pK′7 calculations of Glu286 in ref. 33, we observed that the two models with better cavity hydration, ; 1M56+9w and ′**F** (see Fig. 2 and 3c), yielded similar pK′7 values for Glu286. As shown in Fig. 4, the PMFs for these two models are indeed similar, further supporting the microscopic QM/MM pK′7 calculations, as well as providing the lower bound for the energetics of transferring a proton from Glu286H to PRDa_3_^(–)^. However, even these models feature a barrier of 17–18 kcal mol^–1^ for protonating PRDa_3_^(–)^ and a negligible barrier for the proton flowing back to Glu286^–^.

Hence, our detailed investigation of the energetics of the Glu286H → PRDa_3_^(–)^ proton transfer leads us to conclude that loading of PRDa_3_ by deprotonating Glu286H is not a thermodynamically viable process. The possibility that the BNC can receive a proton before the PLS can also be ruled out since the major part of the barrier in the different PMFs arises from placing a proton in the hydrophobic cavity after deprotonating Glu286; moreover, as calculations below indicate, the p*K*_a_ of PRDa_3_ and BNC are rather similar when heme a is reduced. Hence, the origin for the large free energy penalty seems to be the high pK′7 of Glu286, supported by calculations in ref. 33, which indicated that the pK′7 of Glu286 could not be lowered to the experimental range unless PRDa_3_ is protonated (which is accompanied by a rise in the solvation of Glu286).

### Loading PRDa_3_ through concerted proton transfers and an excess proton in the D-channel is energetically feasible

Since results discussed in the last subsection indicate that loading of the PLS by de-protonating Glu286H is unfavorable, in qualitative agreement with the MEP analysis of Siegbahn and Blomberg^22,23^ using cluster models, we investigate an alternative mechanism in which Glu286 loses and receives a proton at the same time, giving rise to a transiently populated [HGluH]^+^ species. The idea of a “concerted proton transfer” pathway was discussed by both Warshel *et al.* ^17,85^ and by Siegbahn and Blomberg.^22,23^ The key idea was that this mechanism features the movement of a net charge, rather than a dipole as in the process of proton transfer from Glu286H to PRDa_3_^(–)^, thus the coupling of PLS loading to heme a reduction is expected to be stronger. The free energy profile for the underlying process, however, has not been evaluated with a microscopic model.

**Fig. 5:**
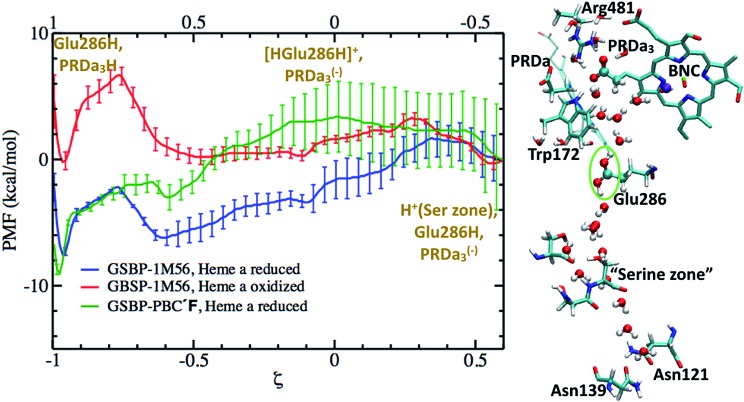
Computed PMFs for the concerted proton transfer mechanism that initiates from the “Serine zone” to PRDa_3_^(–)^ *via* a transiently doubly-protonated Glu286 using different enzyme models and heme a oxidation states. The top *X* axis corresponds to the PMF for the **′F** model while the lower *X* axis corresponds to the ; 1M56 PMFs. On the right, a snapshot illustrates the transiently populated [HGluH]^+^ species in the ; 1M56 model. For a snapshot with a hydrated proton in the “Serine zone”, see Fig. S16c.† Fig. 3a and c illustrate the hydration level in these models.

This concerted mechanism corresponds, in our notation, to the conversion from **P**_R_ to **P**′′R. The **P**′′R state, like the **P**′R and ′**F** states, is also characterized by a large and well hydrated cavity in PBC simulations.^33^ The PMF computation for the concerted proton transfer is initiated from an excess proton in the “serine zone”^44^ and ultimately leads to the loading of PRDa_3_^(–)^ (see Fig. 5); the proton transfer from the entrance of the D-channel to the “serine zone” is discussed in a separate subsection below. With the ; 1M56 model, the results indicate that while the free energy profile is almost flat when heme a is oxidized, the PMF is largely downhill when heme a is reduced. The ′**F** model with a reduced heme a shows a PMF with similar overall exothermicity, although somewhat different energetics are seen for intermediate *ζ* values. Hence, the PMF results explicitly show that the “concerted” proton transfer mechanism is thermodynamically as well as kinetically feasible for loading the putative PLS, PRDa_3_, more so (by ∼8 kcal mol^–1^) when heme a is reduced. This favorable nature of the proton transfer is in qualitative agreement with previous EVB studies of Warshel and co-workers^17,85^ and also the minimum energy path results of Blomberg *et al.* ^23^ (however, see discussion in ESI†). The observation of a doubly protonated Glu286 species in the PMF calculations (see Fig. 5) is, however, unique and has not been considered in previous studies, demonstrating the value of using a general-purpose QM/MM potential function. On the other hand, we note that the doubly protonated Glu286 is a transient species.

### PRDa_3_ flexibility is essential to “kinetic gating”

The fact that the concerted proton transfer mechanism is energetically much more favorable than a “direct” proton transfer from Glu286H to PRDa_3_^(–)^ is consistent with the idea that the proton donor, an excess proton in the D-channel, is much more acidic than a GluH in a hydrophobic region of the protein (see Fig. 1b and additional discussions below). However, an important question for the concerted proton transfer mechanism is that once the excess proton is transferred into the hydrophobic cavity, what is the mechanism that favors PLS loading prior to the protonation of BNC?

**Fig. 6:**
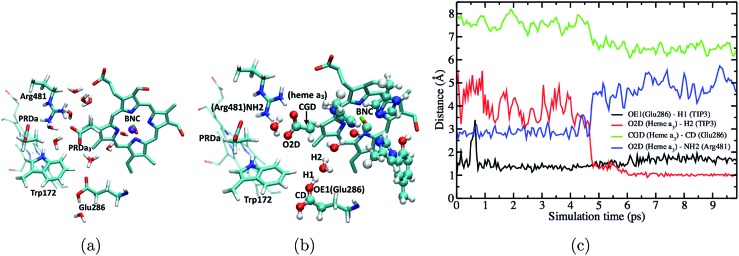
Fast downward rotation of PRDa_3_ in the pre**P′′R** simulations as the proton is transferred into the poorly hydrated hydrophobic cavity. (a) Snapshot at the end of a 50 ns long PBC simulation of the pre**P′′R** state (b) starting from the snapshot represented in (a) and after equilibration with [HGlu286H]^+^ with QM/MM-GSBP; note the intact Arg481-PRDa_3_ salt bridge (c) plot of different distances *versus* time in a QM/MM-GSBP simulation starting from the snapshot represented in (b) and free of any constraints on the position of the excess proton.

Important clues come from the simulations based on the pre**P**′′R model, which is prepared using a PBC simulation with an excess hydronium in the hydrophobic cavity with a low level of hydration. QM/MM simulations reveal a prominent “downward” rotation of PRDa_3_^(–)^ to accept a proton from [HGluH]^+^ *via* a single water molecule (see Fig. 6), thus potentially “snatching” away the proton before it can reach the BNC, which is separated from the excess proton by another water molecule. MD simulations in the pre**P**′′R model starting with an intact Arg481-PRDa_3_ salt bridge suggest that there is no barrier to the “downward” rotation of PRDa_3_^(–)^ as soon as the water molecule closest to Glu286 receives a proton from the doubly protonated Glu286. The insignificant barrier for the protonation of PRDa_3_ in the downward orientation is confirmed by multiple independent simulations with different QM region sizes which include/exclude Cu_B_ with its ligands and Tyr288; in these simulations, PRDa_3_^(–)^ rotates “down” within the first 5 ps to take the proton from the doubly protonated Glu286 through an intervening water (see Fig. 6, note the red trace indicates that the excess proton ends up on PRDa_3_ in the trajectory).

**Fig. 7:**
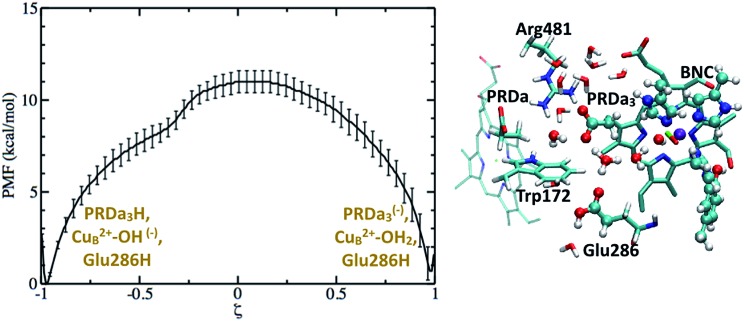
Computed PMF for proton transfer from PRDa_3_H to the OH^(–)^ ligand bound to Cu_B_^2+^ in the pre**P′′R** model; Glu286 remains singly protonated and charge-neutral throughout. The snapshot (*ξ* = –0.4) illustrates the downward rotation of PRDa_3_ to snatch away the proton without barrier once it enters the poorly hydrated cavity. For additional snapshots, see Fig. S12.†

To quantify the competition between PRDa_3_ and BNC for the excess proton, we compute the PMF for the proton equilibration between these two proton accepting groups in the pre**P**′′R model (with heme a reduced). Fig. 7 shows that while PRDa_3_ and the BNC have similar affinities for the proton, proton equilibration between them has a significant barrier of ∼12 kcal mol^–1^. Moreover, the configuration discussed above in which the proton is just transferred from [HGluH]^+^ to a neighboring water in the cavity corresponds to a *ζ* value of –0.4 (see Fig. 7); while the PMF is strictly *downhill* for the proton transfer to the PRDa_3_^(–)^, it is ∼3 kcal mol^–1^ uphill for the proton transfer to the BNC. Therefore, we witness that the conformational flexibility of PRDa_3_ seems essential to the “kinetic gating” phenomena: once the excess proton is transferred into the poorly hydrated cavity, PRDa_3_^(–)^ is able to break away from Arg481 without any significant barrier to rotate downwards and the subsequent protonation of PRDa_3_ is also barrierless. Once PRDa_3_ is protonated, there is a significant barrier for the proton to “leak” to the BNC (Fig. 7).

### Potential roles of PRAa_3_ in preventing proton back flow

Based on the results discussed so far and those in ref. 33, a tentative proton pumping model is that with heme a reduced, PRDa_3_ gets protonated first *via* a concerted proton transfer mechanism, following which the cavity expands and Glu286H donates its proton to the BNC, leading to the ′**F** state with a deprotonated Glu286. This is the state which is most vulnerable to proton backflow, *i.e.*, the proton on PRDa_3_ can fall back to the deprotonated Glu286. In fact, the PMF shown in Fig. 4 indicates that proton back flow in a well hydrated cavity tends to be very favorable with a negligible barrier.

**Fig. 8:**
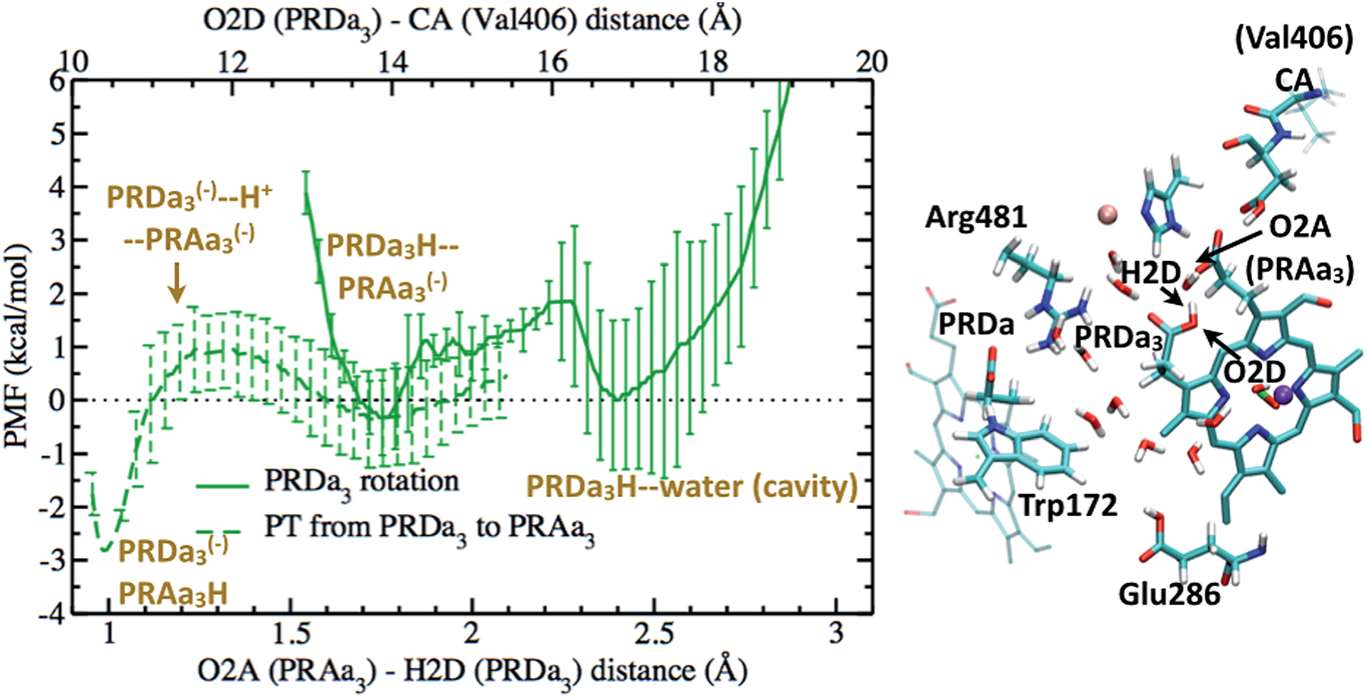
The full green curve represents the PMF for rotation of a protonated PRDa_3_ away from the cavity towards PRAa_3_^(–)^ in the **′F** model, after it has been loaded by a concerted mechanism; the upper *X*-axis labels the corresponding reaction coordinate. The dashed green curve represents the PMF for proton transfer from PRDa_3_H to PRAa_3_^(–)^ after PRDa_3_H becomes directly hydrogen-bonded to PRAa_3_^(–)^ (see the snapshot); the lower *X*-axis labels the corresponding reaction coordinate. Additional snapshots are in Fig. S13 and 14.†

To avoid the back flow, it has been proposed that the negatively charged Glu286 quickly rotates downwards to prevent it from accepting any protons from the cavity; this was, however, not supported by our calculations^32^ (also see discussion in ESI†). Hence a possible alternative is that the loaded proton is no longer on PRDa_3_ in the ′**F**-state (implying that it needs to be transported away from PRDa_3_ during the **P**′′R → ′**F** transition). Indeed, with the ′**F** model (which has a cavity hydration level similar to that in the **P**′′R state^33^), it is found (see Fig. 8) that PRDa_3_H can cross a small barrier of ∼2 kcal mol^–1^ to rotate away from the cavity and share its proton with PRAa_3_^(–)^ (see Fig. S13† for different possible PRDa_3_ orientations).

We also compute the PMF for proton transfer between PRDa_3_ and PRAa_3_, and the results indicate that proton localization on PRAa_3_ is only more favorable by ∼2 kcal mol^–1^ (Fig. 8). Therefore, the rotation of PRDa_3_H and subsequent proton donation to PRAa_3_ alone is not an energetically robust gate. Even if the proton has been transferred to PRAa_3_ before the formation of the ′**F** state, it will be quite easy for it to fall back to PRDa_3_ and ultimately to Glu286 in the ′**F** state. However, an interesting observation, represented in Fig. S14,† is that a protonated PRAa_3_ does not just remain H-bonded to PRDa_3_^(–)^ but can sample a wide variety of conformations, opening up pathways for further conduction of the loaded proton away from PRAa_3_. In the absence of available experimental data on possible proton transfer pathways beyond PRAa_3_ (though see discussion below), it can be suggested that besides providing a favorable mechanism for protonating PRDa_3_, the “concerted” mechanism also makes it favorable for PRDa_3_H to rotate away from the cavity (since Glu286 is protonated) and transfer its proton to PRAa_3_, which is transferred elsewhere towards the P-side in the time-scale for protonation of BNC by Glu286H (this proton transfer most definitely occurs *via* a non-negligible barrier due to the p*K*_a_ of Glu286 still being close to 10). It should be noted that after PRDa_3_ has been loaded by a concerted mechanism in the pre**P**′′R model, cavity opening and water penetration takes place at the time scale of nanoseconds^33^ while the barrier for backflow of the loaded proton back to the cavity and possibly to the BNC is ∼12 kcal mol^–1^ (Fig. 7). Hence, during this process of rise in hydration level in the cavity, the proton can still safely remain on PRDa_3_. After the cavity is better solvated, it is kinetically much more favorable for PRDa_3_H to rotate “up” towards PRAa_3_^(–)^ by crossing a low barrier of around 2 kcal mol^–1^ than to lose the proton back to the cavity (backflow barrier of >10 kcal mol^–1^ in the ′**F** model, shown by the green curve in Fig. 5).

### Bottleneck of PLS loading is likely near the entrance of the D-channel

Experimental studies showed that loading of the PLS (*i.e.*, prior to the protonation of BNC) occurs with a timescale of ∼150 μs (ref. 13) which corresponds to a rate-limiting barrier of ∼12 kcal mol^–1^. However, the concerted proton transfer mechanism that starts with an excess proton in the “serine zone”, especially with heme a reduced, predicts a downhill loading of PRDa_3_ (Fig. 5), suggesting that the bottleneck of PLS loading is located elsewhere. A candidate site is the pair of Asn residues (Asn121 and Asn139) at the N-side entrance of the D-channel, close to Asp132, which transfers protons picked up from the bulk to the D-channel. Several mutation studies of Asp132, Asn121 and Asn139 have been carried out and revealed that even certain charge-neutral mutations lead to decoupling of proton pumping and chemical activity.^93–99^ Hence it is possible that the region around these residues plays an important role in the rate of proton uptake and forms the bottleneck of proton pumping sub steps.

Examination of the water configurations in the crystal structure and previous MD simulations^100^ indicate that the pair of Asn side chains need to rotate to let a proton (or hydronium) to pass. This significantly complicates the calculation of proton transfers and requires using more complex reaction coordinates beyond mCEC (see ESI†). To gain insights into proton transfer activity through this constriction region, we carry out an *in silico* mutation of Asn121 and Asn139 to serine residues such that the polar nature of the region is maintained while steric effects associated with the Asn side-chains can be minimized. In the study by Pomes and co-workers,^100^ the barrier for the rotation of the Asn139 side-chain from a “closed” to an “open” configuration was found to be ∼4 kcal mol^–1^. Hence, proton transfer calculations for the N139S/N121S mutant can be taken as a reasonable model for obtaining an estimate for the barrier of transferring a proton through this region.

As shown in Fig. S15,† the barrier for proton transfer from Asp132 to the “serine zone” is found to be ∼16 kcal mol^–1^, with the bottleneck region corresponding to the passage of the proton *via* the constricted region between Ser121 and Ser139 (Fig. S16†). Although this barrier is higher than the value of ∼12 kcal mol^–1^, considering the classical nature of the nuclear dynamics^101^ and relative proton affinity errors (∼2 kcal mol^–1^) associated with the DFTB3 variant used here as QM^77^ (see Methods), this result provides a possible explanation for the ∼150 μs time-scale observed experimentally.

## Discussion

In the following, we first summarize the findings from this study and their implication to the proton pumping mechanism in CcO, then we discuss the connection between these results with experimental data. For comparison with previous computational studies and a discussion of remaining mechanistic issues, see ESI.†

### A proposal for proton pumping steps in CcO – fundamental differences between concerted and step-wise mechanisms

**Fig. 9:**
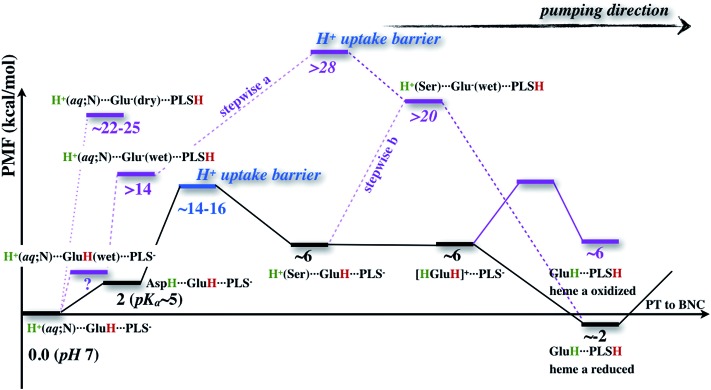
A schematic free energy profile associated with the proton transfer processes studied here, highlighting the bottleneck for the overall loading process of the PLS (assumed to be PRDa_3_), the importance of heme a reduction, and the difficulty associated with directly loading PLS with a neutral Glu286 (dotted line), even with a “stepwise” mechanism (dashed line) in which the deprotonated Glu286 in a hydrated cavity is quickly reprotonated (with the proton transfer from Glu286H to PLS either before (stepwise a) or after proton (stepwise b) uptake in the D-channel). The free energy values in normal text are based on PMFs computed in this work (adjusted with the ∼2 kcal mol^–1^ proton affinity errors for acetic acid and small water clusters), and values in italics are estimated by assuming that proton uptake in the D-channel is independent from the proton transfer from GluH286 to PRDa_3_. In the “stepwise” mechanism, the free energy cost of hydrating the hydrophobic cavity prior to any proton transfer is not included.

**Fig. 10:**
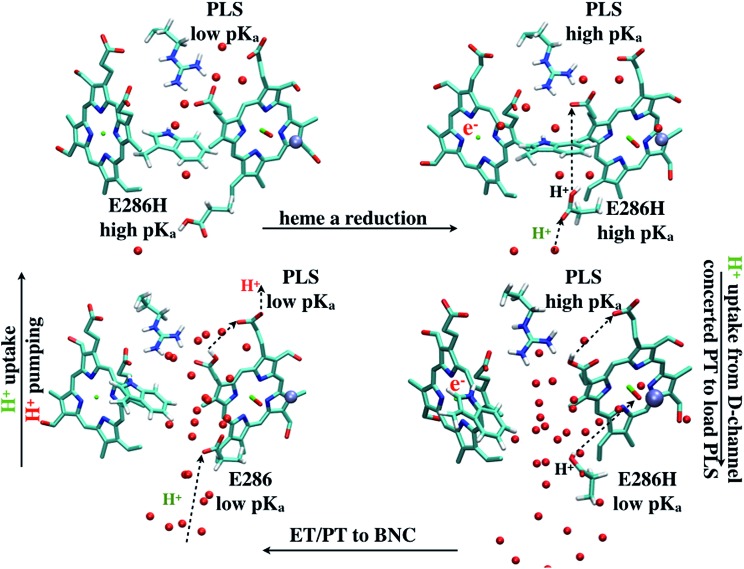
A scheme (revised based on ref. 33) that illustrates how change of hydration level in the hydrophobic cavity coupled to a concerted proton transfer to PRDa_3_ drives the proton pumping cycle in CcO. This mechanism allows the loading of PRDa_3_ without involving a deprotonated Glu286 in a poorly hydrated hydrophobic cavity. Once the PRDa_3_ is protonated, the hydration level of the cavity increases,^33^ which lowers the p*K*_a_ of Glu286, allowing it to transfer the proton to the BNC.

The underlying free energy diagram and the schematic pumping mechanism that emerged from our current and previous^33^ work are summarized in Fig. 9 and 10, respectively. As discussed below, the mechanism features several somewhat surprising findings, although they are, at hindsight, straightforward to rationalize on physical grounds. Overall, the proposed mechanism lends new supports to the “constraints” that emerged from phenomenological analysis of proton pumping in CcO^28^ with structural and energetic details.

We find it is energetically very unfavorable to deprotonate Glu286H in a **P**_R_ like state and transfer the proton to PRDa_3_^(–)^, the tentative PLS that most likely needs to be at least transiently loaded. With a low level of hydration, the proton transfer is uphill by more than 20 kcal mol^–1^ (Fig. 4). Although this is in contrast with most common assumptions in the experimental literature (though the possibility was raised in previous computational studies,^17,22,23^ see additional discussion in ESI†), the underlying physical picture is fairly simple: the protein does not like to “trade” a negative charge next to an Arg (PRDa_3_^(–)^) for a negative charge in a hydrophobic environment (Glu286^(–)^). While an increased solvation of Glu286^(–)^ makes the proton transfer more favorable, this exchange of the location of a negative charge is still highly unfavored by the protein microenvironment. The lower bounds for the proton transfer barrier/endothermicity are found using a ; 1M56+9w model with scaled charges of Arg481 and the ′**F** model, both with heme a reduced, which still lead to PRDa_3_H to be ∼14 kcal mol^–1^ less stable than Glu286H, with a minute barrier (∼3–4 kcal mol^–1^) for proton backflow (Fig. 4).

The alternative mechanism thus involves a concerted proton transfer that starts with a “metastable” excess proton in the serine zone of the D-channel. There are several reasons that this mechanism features more reasonable energetics and is attractive from a functional perspective. Foremost, due to the concerted nature, Glu286H is not deprotonated during the proton transfer process; in fact, it is transiently doubly protonated (Fig. 5). Therefore, the high pK′7 of Glu286 in a **P**_R_ like state, which features a low level of hydration in the cavity region,^33^ does not hinder the proton transfer and thus loading of the PLS. Moreover, as originally recognized by Siegbahn and Blomberg,^22,23^ the concerted proton transfer mechanism involves the motion of a net positive charge, rather than a dipole as in the case of proton transfer between Glu286H and PRDa_3_^(–)^. As a result, the coupling of PLS loading and heme a reduction is much stronger in the concerted mechanism; this is borne out by the calculations in this work: while the effect of heme a reduction on the Glu286H → PRDa_3_^(–)^ transfer is less than 4 kcal mol^–1^ (Fig. 4), PRDa_3_^(–)^ loading *via* the concerted proton transfer process becomes 8 kcal mol^–1^ more favorable with a reduced heme a (Fig. 5).

Our mechanism provides new clues to how branching (timing) of proton transfers to the BNC and PLS is modulated. With a rather dry cavity in the **P**_R_ like state, proton transfer into the cavity *via* the concerted mechanism attracts the PRDa_3_ to rotate “downwards” into the cavity, thus snatching the excess proton away without any significant barrier before the latter has a chance to migrate towards the BNC (Fig. 7). Loading of the PRDa_3_ then induces cavity expansion and increase of the local hydration level, which in turn helps lower the pK′7 of Glu286, opening the gate for the subsequent proton transfer from Glu286H to the BNC. Therefore, conformational flexibility of PRDa_3_ and the coupling among PRDa_3_ protonation, cavity hydration level and Glu286 pK′7 form the basis of “kinetic gating” that appears to underline the pumping efficiency of CcO.^28^

Is the concerted mechanism really distinct from a “stepwise” mechanism in which Glu286H donates its proton to PRDa_3_ and then gets reprotonated quickly? For example, Fig. 4 indicates that with a hydrated cavity, the proton transfer from Glu286H to PRDa_3_ has a barrier of about 18 kcal mol^–1^, which appears to be not too far from the rate-limiting barrier for the concerted mechanism, which implicates the uptake of the excess proton through the D-channel. Therefore, it is tempting to suggest that a “stepwise” mechanism would also work, where a conformational change (prior to any proton transfers) alters the hydration level of the cavity, which makes it less unfavorable to transfer the proton from Glu286H to PRDa_3_; once PRDa_3_ is protonated, the expanded and better hydrated cavity is stabilized, and finally the deprotonated Glu286 gets quickly reprotonated.

The flaw in this argument is that it does not consider the proton uptake energetics for the Glu286 reprotonation. Since Glu286 is deeply buried in the protein interior, the energetics and kinetic bottleneck for the proton uptake should not be sensitive to the protonation state of Glu286 (see Fig. S17 in the ESI†). Once these are taken into consideration, as illustrated in Fig. 9, regardless of whether the proton uptake takes place before (stepwise (b)) or after (stepwise (a)) the proton transfer from Glu286H to PRDa_3_, the rate-limiting barrier for the “stepwise” mechanism is much higher than the concerted one. Again, the key difference is that the concerted mechanism does not involve a deprotonated Glu286, which is a high free energy species unless PRDa_3_ is loaded.

### Connection to experimental studies

Taking the intrinsic error bars of our QM/MM protocol, such as the proton affinity error for the donor/acceptor groups (typically <2 kcal mol^–1^ for the proton transfers considered here), into consideration, the energetics for our proposed mechanism are in line with available experimental data. As discussed above, the highest barrier estimated from our calculations (∼16 kcal mol^–1^) is located at the entrance of the D-channel and a few kcal mol^–1^ higher than the experimental estimate of 12.4 kcal mol^–1^ (150 μs); this can be considered a fair agreement since the calculated barrier does not include nuclear quantum effects, which would enhance the rate of transfer, although it's important to bear in mind that the barrier is calculated for the N139S/N121S mutant due to technical reasons.

The location of the rate-limiting barrier underlines the significance of that region in the proton pumping cycle; this is in line with the observation that mutations in this region, even charge-neutral mutations, often lead to major impacts on the proton pumping activity,^93–99^ although a molecular level understanding of the mutation effects remains elusive (see ESI†). We note that the 150 μs time scale is for PLS loading and not for the protonation of the BNC;^13^ it is likely that BNC protonation (thus **F** formation) has a significant barrier since Glu286 in the hydrated cavity still has a rather high p*K*_a_ ∼ 10. Thus the experimental observation that the rate of **F** formation is not substantially altered in the D132N mutant,^102^ in which proton uptake through the D-channel is blocked, is not against a significant barrier for proton uptake through the D-channel. Moreover, it is worthwhile noting that the D132N mutants exhibit abnormal respiratory control ratios (RCRs) – *i.e.*, their activities are inhibited rather than stimulated by the electrical gradient;^45,102^ the interpretation is that protons leak through the exit channel to support the low level of enzyme turnover.^103^

The p*K*_a_ values for a few groups have been estimated based on available experimental kinetic data; they are ∼9.4 for Glu286, ∼9 for the PLS when heme a is reduced and ∼5 for the PLS when both electron and proton transfer to the BNC have taken place.^104^ The issue of Glu286 p*K*_a_ has been discussed in detail in our previous studies^31,33^ and therefore won't be elaborated on further here. Using the solution reference of pH 7, the free energy diagram in Fig. 9 would indeed imply an effective p*K*_a_ > 7 for PRDa_3_ when heme a is reduced (loading of PRDa_3_^(–)^ is slightly exothermic relative to solution) and ∼5 p*K*_a_ unit lower when heme a is oxidized (loading of PRDa_3_^(–)^ is +8 kcal mol^–1^ more endothermic).

The concerted proton transfer mechanism features Glu286 as both a proton relay (during PLS loading) and a proton donor (for BNC protonation) group; the relay function can, in principle, be accomplished with other polar groups (*e.g.*, water, His or Ser) while the proton donation to BNC apparently requires only a modest p*K*_a_ (∼9–10, which is accessible to Tyr). In other words, the concerted proton transfer mechanism imposes, in fact, fairly weak “constraint” on the residue at the boundary of the active site. This is qualitatively consistent with the experimental observation for CcO from *Paracoccus denitrificans*:^105^ while replacement of this glutamic acid and a conserved glycine nearby lowers the catalytic activity to <0.1% of the wild-type value, if, in addition, a nearby phenylalanine is changed to tyrosine, the activity rises more than 100-fold and proton translocation is restored. In other words, a glutamate is not indispensable for the CcO function, and a polar protic amino acid close to the cavity region is sufficient. In fact, in some families of heme-copper oxygen reductases, the D-channel and glutamate do not appear to exist and proton uptake proceeds through a channel analogous to the K-channel in the A-family of heme-copper oxygen reductases (*e.g.*, the *R. sphaeroides* CcO discussed here); *e.g.*, the channel in the *T. thermophilus* oxidase features largely serines and tyrosines,^106^ which would have side chain p*K*_a_ values around 10 or higher. Infrared spectroscopy studies found evidence for the deprotonation cycle of Glu286 during the functional cycle,^107^ although these observations are not directly contradictory to our finding because the Glu does give its proton to the chemical site in our mechanism.

In the free energy diagram, the configurations that correspond to having an excess proton in the serine zone correspond to a fairly flat region (Fig. 5 and S15†) rather than a major thermodynamic trap.^44,45^ Therefore, this “metastable state” is not kinetically significant. Nevertheless, if serine residues in this region are mutated into hydrophobic ones, it is expected that the excess proton is no longer stabilized and thus the loading of the PLS gets perturbed; experimentally,^45^ it was observed that both the Ser200Ile and Ser200Val/Ser201Val variants maintained the ability to pump protons, although with slowed oxidation kinetics for the **P**_R_ → **F** and **F** → **O** transitions.

Our discussion regarding both PRDa_3_ and PRAa_3_ being implicated as PLS is consistent with the experimental results of Gennis and co-workers.^108,109^ They found that while mutating Arg481 (which forms a salt-bridge with PRDa_3_) to a hydrophobic residue does not completely abolish pumping, mutating the conserved Asp hydrogen bonded to PRAa_3_ leads to a decoupling phenotype. These observations do not directly argue against the importance of PRDa_3_ (since it remains flexible and titratable), but they emphasize that pumping relies on the ability to transfer the proton to PRAa_3_ and beyond, as we discussed above in light of the computational results.

In terms of predictions that our mechanism may lead to, there are several considerations. First, as mentioned in ref. 33, the expansion of cavity (by ∼150 Å^3^) and increase of hydration level upon protonation of PRDa_3_ are reproducible in independent MD simulations. Change of internal volume and hydration of such magnitudes should be detectable with appropriate experimental techniques, such as photo acoustic and infrared spectroscopies, respectively. Since the cavity expansion is due largely to the displacement of a loop that bears Trp172, rigidifying that loop by substituting the conserved glycines would then likely lead to significant impact on the pumping activity; along this line, it is worth noting that the mutation of a Gly in this loop (Gly171) to Asp was shown to lead to CcO malfunction.^6^ Second, our mechanism underlines the significance of conformational flexibility of PRDa_3_, without which the proton transferred into the cavity may partition rather equally between PRDa_3_ and the BNC, even with heme a reduced. Therefore, infrared studies with isotopically labeled propionates should provide evidence for the conformational transitions of PRDa_3_, and if feasible, incorporating heme with shorter carboxylate chains is predicted to lead to reduced pumping. Finally, since many mutations of residues at the mouth of the D-channel have led to significant impact on the proton pumping activity, it would be valuable to evaluate the activity of the specific double mutant (N139S/N121S) studied here and confirm that it is functionally active.

## Concluding remarks

Proton pumping is one of the most fascinating processes in bioenergy transduction. With numerous experimental and computational studies, it is now clear that different strategies are used in different types of proton pumps.^9^ Among them, the most poorly understood class is represented by cytochrome c oxidase (CcO), which drives proton pumping with great efficiency using the energy released by oxygen reduction to water. Despite immense efforts, many fundamental questions regarding the mechanism that governs the vectorial nature of the proton transport in CcO remain to be answered. For example, although elegant kinetic network analysis^28,41^ and other arguments^24,40^ emphasized the importance of kinetic constraints to an efficient transport, the microscopic basis for such “kinetic gating” principles has not been elucidated. Previous molecular simulations have probed different aspects of proton transfers in CcO, especially concerning the potential role(s) of the conserved Glu286, but no consensus is reached regarding how Glu286 controls the branching (or timing) of proton transfers to the chemical site (the binuclear center, BNC) and the proton loading site (PLS), which is an important aspect of kinetic gating.

In this study, motivated in part by our recent finding^33^ that the hydrophobic cavity of CcO undergoes a significant change in the level of hydration depending on the protonation state of the tentative PLS, the propionate D of heme a_3_, we have carried out extensive QM (DFTB3)/MM free energy simulations to probe the proton transfer mechanisms in CcO. The most essential finding of our study is that the loading of PLS requires a concerted process in which Glu286H delivers the proton to PRDa_3_ while being reprotonated by an excess proton coming from the D-channel, in qualitative agreement with the MEP analysis based on cluster models by Siegbahn and Blomberg.^22,23^ The concerted nature is favorable because it avoids having a deprotonated Glu286 in a rather poorly hydrated region of the protein; by contrast, a “stepwise” pathway in which Glu286H first transfers the proton to PRDa_3_ and subsequently gets reprotonated in a separate step would be much less favorable (see Fig. 9). As suggested in ref. 33, once PRDa_3_ is protonated, the hydrophobic cavity is better hydrated, lowering the p*K*_a_ of Glu286 to a range appropriate for transferring the proton to BNC. In other words, the concerted proton transfer mechanism builds in a natural sequence for the protonation of the PLS and BNC; our work suggests that the structural flexibility of PRDa_3_ also contributes to the preference of PLS loading prior to the protonation of BNC. Moreover, since a net charge is transferred in the concerted mechanism^22,85^ (rather than a dipole in a stepwise mechanism), loading of PLS is tightly coupled to the reduction of heme a. These two features are precisely the kinetic gating principles underscored by the kinetic network analysis of Kim and Hummer.^28^

The results of our recent^33^ and current studies of CcO emphasize the importance of carefully considering changes in the internal hydration level of proteins and p*K*_a_ of buried residues^19,31,52^ for modulating the timing of proton transfers. These changes may not implicate any major structural changes at the global scale but likely require notable local structural transitions. Therefore, putting seemingly “innocent” structural constraints (*e.g.*, on the C_α_ atoms in a large transmembrane protein) may prevent important changes in the protein interior from being sampled. On the other hand, as illustrated in this work, by studying QM/MM models established using different structural models from unconstrained classical simulations, we are able to gain insights into the role of cavity hydration in proton transfer and set bounds on the proton transfer barriers and thermodynamics. We expect that this strategy is valuable to the analysis of other systems. In the future, an important technical challenge to tackle is to explicitly couple^110^ hydration changes of internal cavities, local structural transitions and proton transfers in multi-dimensional PMF or free energy path calculations so that the causal relationships between these processes of distinct nature can be better revealed.
