# Supplementary material for: Microscopic basis for kinetic gating in cytochrome c oxidase: insights from QM/MM analysis
Source: Chem Sci. 2014 Oct 6;6(1):826–41. doi: 10.1039/c4sc01674b (PMC4321873; doi:10.1039/c4sc01674b)
Supplement: Supplementary file 1 [file SC-006-C4SC01674B-s001.pdf]

Electronic Supplementary Material (ESI) for Chemical Science.
This journal is © The Royal Society of Chemistry 2014

# Supporting Information for: Microscopic basis for kinetic gating in Cytochrome c oxidase: insights from QM/MM analysis

Puja Goyal, Shuo Yang, and Qiang Cui*

*Department of Chemistry and Theoretical Chemistry Institute, University of Wisconsin-Madison, 1101 University Avenue, Madison, WI 53706*

E-mail: cui@chem.wisc.edu

## Additional Benchmark and analysis

### Benchmarks for DFTB3/3OB for the proton affinity of gas phase copper complexes $(Im)_3Cu^{2+}$-$OH_2$ and (4-MePhOH-Im)$(Im)_2Cu^{2+}$-$OH_2$(models of the $Cu_B$ site)

The preliminary parameterization and benchmark of DFTB3 for copper based on an $l$-dependent Hubbard formulation are reported recently.[1] Here, we specifically test the parameterization for two models of the $Cu_B$ site of CcO: (1) $(Im)_3Cu^{2+}$-$OH_2$, in which water and three imidazole (denoted by Im) molecules act as ligands to $Cu^{2+}$ (2) (4-MePhOH-Im)$(Im)_2Cu^{2+}$-$OH_2$, which is different from (1) in that one of the imidazole ligands is cross-linked to 4-methylphenol (denoted by 4-MePhOH), the latter acting as an analog of Tyr288 in CcO which is cross-linked to a histidine ligand of $Cu^{2+}$.

*To whom correspondence should be addressed

As shown in Figs. S1,S2 and Table S1, overall, the DFTB3/3OB optimized structures agree quite well with the UB3LYP minimized structures, in terms of Cu-ligand distances and angles. However, for $(Im)_3Cu^{2+}$-$OH_2$, the water ligand is significantly displaced in the DFTB3 structure compared to that from UB3LYP, with a much longer Cu-O distance of 2.36 Å with DFTB3 as opposed to 2.09 Å with UB3LYP; the imidazole ligands also feature somewhat different orientations, especially in the hydroxide-bound state of copper. The slight deviation of imidazole ligands is less of a concern in the context of protein simulations since the corresponding His ligands are conformationally constrained by the protein structure. The complexes with a cross-linked 4-methylphenol have UB3LYP and DFTB3 optimized geometries in much better agreement with each other, although the only atom frozen in space during the optimizations is the methyl carbon atom of 4-methylphenol.

Despite differences in optimized geometries, the PAs of $(Im)_3Cu^{2+}$-$OH_2$ from UB3LYP and DFTB3 are within ∼1 kcal/mol of each other (Table S2). The UB3LYP//DFTB3 and DFTB3//UB3LYP calculations indicate that the difference in the PAs with UB3LYP and DFTB3 are possibly due to the difference in $(Im)_3Cu^{2+}$-$OH_2$ geometries predicted by the two methods. Inclusion of the cross-linked 4-methylphenol reveals a larger error, on the order of 4 kcal/mol, in the PA predicted by DFTB3. While Tyr288 is included in the QM region in the calculation of the PMF for PT between $PRDa_3$ and the BNC (reported in the main text), we note that DFTB3/3OB also has an error of ∼3-4 kcal/mol for the PA of carboxylic acids[2] and hence the relative energies of the minima in the PMF would remain largely unaffected. The configurations which constitute the barrier for proton transfer between $PRDa_3$ and the BNC involve hydronium interacting with $PRDa_3^{(-)}$ and hence would also remain largely unaffected by the 1-2 kcal/mol underestimation of the PA of small protonated water clusters by DFTB3/3OB.[2]

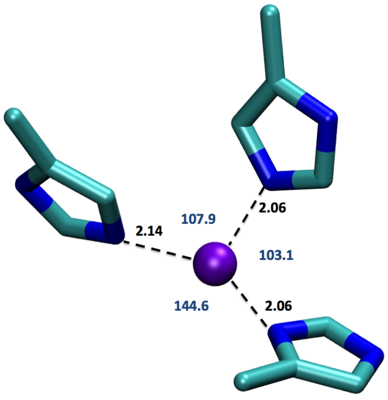


(a) 1M56

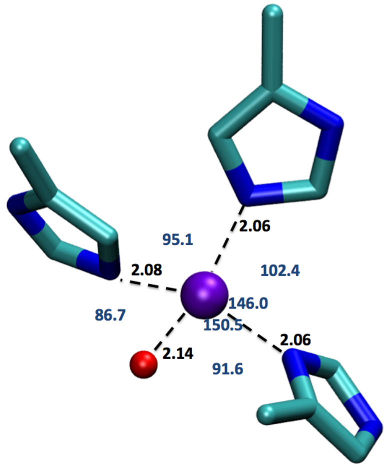


(b) 2GSM

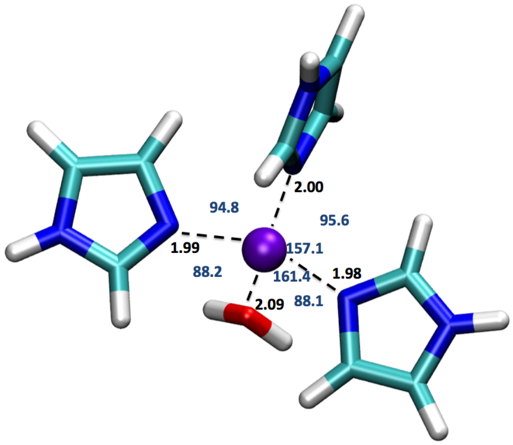


(c) $(Im)_3Cu^{2+}$-$OH_2$

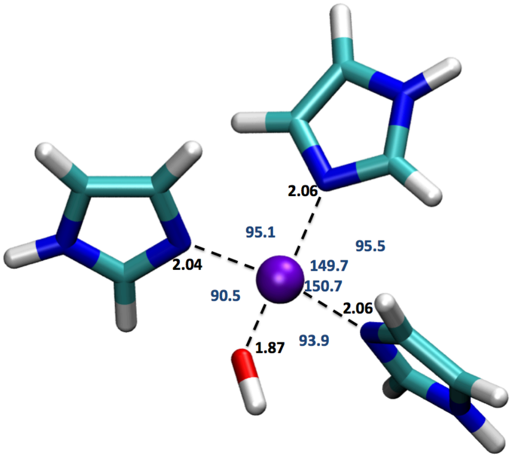


(d) $(Im)_3Cu^{2+}$-$OH^{(-)}$

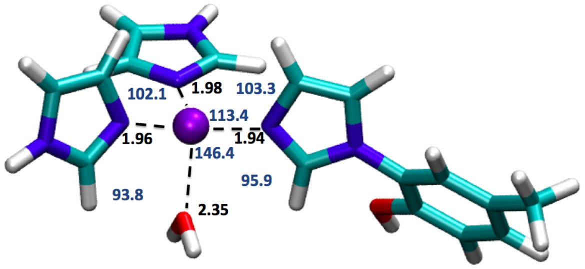


(e) (4-MePhOH-Im)$(Im)_2Cu^{2+}$-$OH_2$

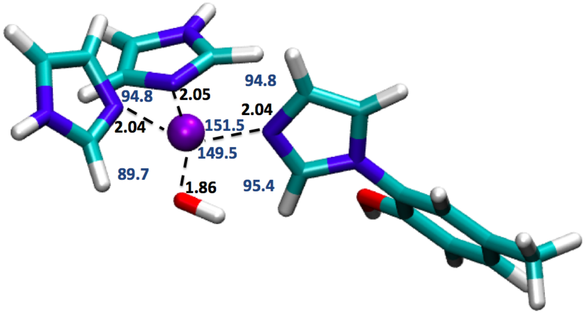


(f) (4-MePhOH-Im)$(Im)_2Cu^{2+}$-$OH^{(-)}$

Figure S1: (a) and (b): Structure of the $Cu_B$ site (excluding Tyr288 which is cross-linked to a His ligand of $Cu_B$) in two oxidized state crystal structures of CcO, 1M56 and 2GSM (the latter with a resolved oxygen bound to Cu). (c), (d), (e) and (f): UB3LYP optimized structures of gas-phase models based on the $Cu_B$ site in CcO, with water or $OH^{(-)}$ as the oxygenous ligand bound to Cu. (c) and (d) are optimized with the 6-311+G(d,p) basis set while (e) and (f) are optimized with the 6-31+G(d,p) basis set. During the optimization of (e) and (f), the methyl C atom of 4-MePhOH is frozen in space. The Cu-ligated atom distances are shown in black numbers and are in units of Å. Various angles involving the metal and ligated atoms are shown in blue numbers and are in units of degree. The angles larger than 140° are between "diagonally opposite" ligands.

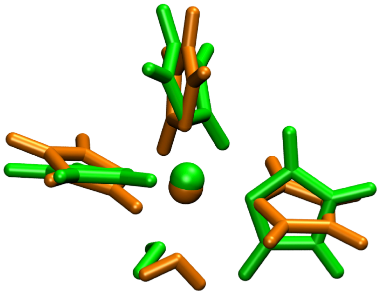

(a) $(Im)_3Cu^{2+}$-$OH_2$

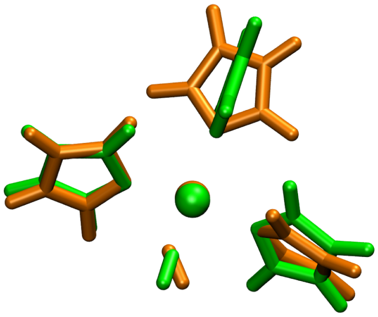

(b) $(Im)_3Cu^{2+}$-$OH^{(-)}$

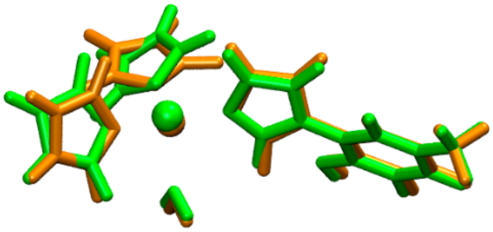

(c) (4-MePhOH-Im)$(Im)_2Cu^{2+}$-$OH_2$

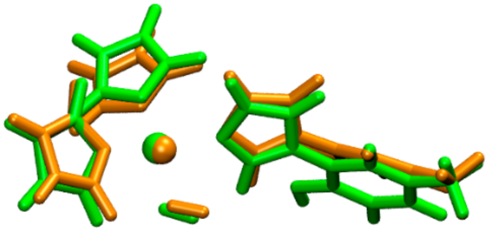

(d) (4-MePhOH-Im)$(Im)_2Cu^{2+}$-$OH^{(-)}$

Figure S2: Overlay of UB3LYP (in orange) and DFTB3/3OB (in green) optimized structures for gas-phase Cu complexes modeled on the $Cu_B$ site in CcO. (a) and (b) are optimized with the 6-311+G(d,p) basis set while (c) and (d) are optimized with the 6-31+G(d,p) basis set. During the optimization of (c) and (d), the methyl C atom of 4-MePhOH is frozen in space.

Table S1: Comparison of Cu-ligated atom distances and various angles involving Cu and its ligated atoms in UB3LYP[a] and DFTB3/3OB optimized structures of $(Im)_3Cu^{2+}$-$OH_2$, $(Im)_3Cu^{2+}$-$OH^{(-)}$, (4-MePhOh-Im)$(Im)_2Cu^{2+}$-$OH_2$ and (4-MePhOh-Im)$(Im)_2Cu^{2+}$-$OH^{(-)}$.

| | $(Im)_3Cu^{2+}$-$OH_2$ | | $(Im)_3Cu^{2+}$-$OH^{(-)}$ | | (4-MePhOH-Im)$(Im)_2Cu^{2+}$-$OH_2$ | | (4-MePhOH-Im)$(Im)_2Cu^{2+}$-$OH^{(-)}$ | |
|---|---|---|---|---|---|---|---|---|
| | UB3LYP | DFTB3 | UB3LYP | DFTB3 | UB3LYP | DFTB3 | UB3LYP | DFTB3 |
| $r_{Cu-N_1}$ (Å) | 1.98 | 1.92 | 2.06 | 2.00 | 1.94 | 1.91 | 2.04 | 2.01 |
| $r_{Cu-N_2}$ (Å) | 2.00 | 1.98 | 2.06 | 2.03 | 1.98 | 1.99 | 2.05 | 2.02 |
| $r_{Cu-N_3}$ (Å) | 1.99 | 1.92 | 2.04 | 2.00 | 1.96 | 1.93 | 2.04 | 2.00 |
| $r_{Cu-O}$ (Å) | 2.09 | 2.36 | 1.87 | 1.91 | 2.35 | 2.42 | 1.86 | 1.90 |
| $\theta_{N_1-Cu-N_2}(^0)$ | 95.6 | 100.1 | 95.5 | 95.1 | 103.3 | 105.0 | 94.8 | 95.9 |
| $\theta_{N_1-Cu-N_3}(^0)$ | 161.5 | 156.2 | 150.7 | 154.9 | 146.4 | 151.9 | 149.5 | 150.0 |
| $\theta_{N_1-Cu-O}(^0)$ | 88.1 | 91.1 | 93.9 | 91.7 | 95.9 | 92.7 | 95.4 | 93.8 |
| $\theta_{N_2-Cu-N_3}(^0)$ | 94.8 | 101.1 | 95.1 | 95.1 | 102.1 | 100.5 | 94.8 | 95.4 |
| $\theta_{N_2-Cu-O}(^0)$ | 157.1 | 115.8 | 149.7 | 147.9 | 113.4 | 105.7 | 151.5 | 147.8 |
| $\theta_{N_3-Cu-O}(^0)$ | 88.2 | 89.4 | 90.5 | 91.7 | 93.8 | 91.6 | 89.7 | 91.2 |

[a]UB3LYP geometry optimizations for the $(Im)_3Cu^{2+}$-$OH_2$ and $(Im)_3Cu^{2+}$-$OH^{(-)}$ complexes are performed with the 6-311+G(d,p) basis set while those for the complexes involving MePhOH are carried out with the 6-31+G(d,p) basis set.

Table S2: Proton affinity (PA) in kcal/mol of $(Im)_3Cu^{2+}$-$OH_2$ and (4-MePhOH-Im)$(Im)_2Cu^{2+}$-$OH_2$ (the protonated forms; the deprotonated molecules are $(Im)_3Cu^{2+}$-$OH^{(-)}$ and (4-MePhOH-Im)$(Im)_2Cu^{2+}$-$OH^{(-)}$, respectively) calculated by different methods.

| | UB3LYP[a] | DFTB3[b] | UB3LYP//DFTB3[c] | DFTB3//UB3LYP[d] |
|---|---|---|---|---|
| $(Im)_3Cu^{2+}$-$OH_2$ | 182.6 | 183.9 (+1.3)[e] | 181.9 (-0.7)[e] | 182.5 (-0.1)[e] |
| (4-MePhOH-Im)$(Im)_2Cu^{2+}$-$OH_2$ | 189.1 | 193.4 (+4.3)[e] | 188.7 (-0.4)[e] | 193.7 (+4.6)[e] |

[a]The basis set used is 6-311+G(d,p). [b]Using the 3OB parameter set. [c]UB3LYP/6-311+G(d,p) single-point energy calculation for DFTB3/3OB optimized structure. [d]DFTB3/3OB single-point energy calculation for UB3LYP optimized structure. UB3LYP with the 6-311+G(d,p) basis set is used for the complexes without MePhOH while UB3LYP/6-31+G(d,p) is used for the complexes with the cross-linked MePhOH. [e]Numbers in parentheses are PA(method) - PA(B3LYP).

## Benchmarks for the description of hydrogen-bonding interactions with DFTB/MM

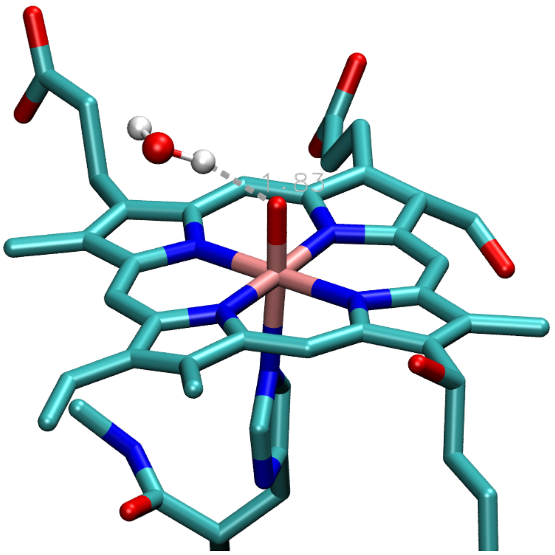


Figure S3: DFTB3/3OB/MM optimized structure of heme $a_3$-water complex. His419, a ligand of heme $a_3$ Fe, has its backbone capped with -$NHCH_3$ and -$COCH_3$ groups. Only the water molecule (shown in CPK) is treated with DFTB3/3OB. The atoms frozen during optimization are Fe, the N atom of His419, which coordinates to Fe, the backbone C$\alpha$ atom of His419, the carboxylate C atoms of the two heme propionates, and C11 and C24 in the tail. The relative positions of the frozen atoms are the same as in the crystal structure (PDB ID 1M56).

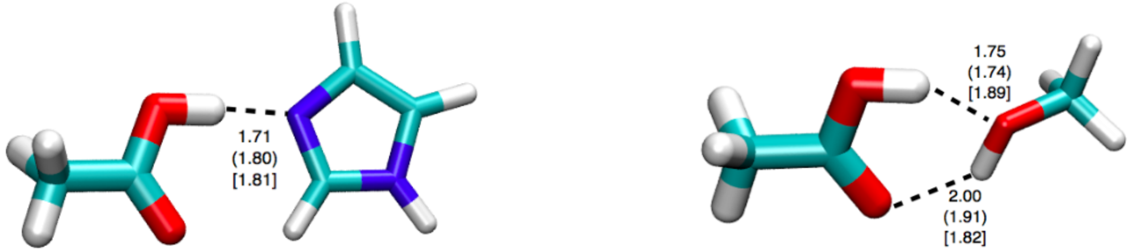


(a) Acetic acid-imidazole

(b) Acetic acid-methanol

Figure S4: B3LYP/6-31+G(d,p) optimized structures of two hydrogen-bonded complexes. The numbers represent hydrogen-bond distances in Å. Bare: B3LYP; within parentheses: DFTB3/3OB; within square brackets: DFTB3/3OB/MM, where the species in bolder licorice is treated with DFTB3/3OB. Distances from other DFTB variants/calculations are not shown since they are very similar to the DFTB3/3OB or DFTB3/3OB/MM distances except that in the case of acetic acid-imidazole, DFTB3/MIO/fit and DFTB3-diag/MIO predict the hydrogen-bond distance to be 1.91 Å and 1.93 Å respectively, as opposed to a distance of 1.80 Å from DFTB3/3OB.

Table S3: Binding energy in kcal/mol (no corrections for basis set superposition error are included)[a]

| | water-heme $a_3$ | acetic acid-imidazole | acetic acid-methanol |
|---|---|---|---|
| MP2/6-311+G(d,p) | | -13.5 | -11.1 |
| B3LYP/6-311+G(d,p) | | -12.4 | -10.5 |
| B3LYP/6-31+G(d,p) | -7.9 | -12.6 | -10.8 |
| DFTB3/3OB | | -8.5 | -9.6 |
| DFTB3/3OB/MM | -8.5 | -12.3 | -10.2 |
| DFTB3/MIO/fit | | -9.0 | -10.2 |
| DFTB3/MIO/fit/MM | -13.3 | -11.9 | -9.9 |
| DFTB3-diag/MIO | | -8.3 | -9.7 |
| DFTB3-diag/MIO/MM | -13.3 | -11.7 | -9.6 |

[a]For water-heme $a_3$, all calculations are single-point calculations on DFTB3/3OB/MM optimized structures. For acetic acid-imidazole and acetic acid-methanol, MP2/6-311+G(d,p) and B3LYP/6-311+G(d,p) single-point calculations are carried out on B3LYP/6-31+G(d,p) optimized structures. For all DFTB calculations, optimizations are carried out at the respective level of theory.

## Comparison of proton transfer energetics from potential of mean force and thermodynamic integration calculations

Figs. S5 and S6 show "direct proton transfer" and "concerted proton transfer" PMFs for different GSBP models for different heme a oxidation states computed using DFTB3-diag/MIO+gaus.[3] Quantitatively, they are different from those presented in the main text (Fig. S5 should be compared to Fig. 4, Fig. S6 should be compared to Fig. 5) because, as stated in Methods, DFTB3[4]/MIO/fit+gaus is used for the results in the main text. They differ slightly in the predicted hydrogen bonding energies, and the largest difference lies in the errors of proton affinity for acetic acid and water clusters; at the DFTB3/MIO/fit+gaus level, the relative errors are about 2 kcal/mol, while it is $\sim$ 7 kcal/mol with DFTB3-diag/MIO+gaus. For the "direct" proton transfer from Glu286H to PRD$a_3$, both donor and acceptor groups are carboxylic acids and therefore the endothermicity of the PMF is not very sensitive to the specific DFTB3 method used. For the concerted proton transfer, the donor is essentially a protonated water cluster while the acceptor is a carboxylic group, thus the exothermicity of the PMF is more sensitive to the DFTB3 method used. Over-

all, however, the trends are similar, highlighting the robustness of the conclusions that the concerted proton transfer is (i) energetically much more favorable than the direct loading of PLS by Glu286H and (ii) coupled more tightly with the reduction of heme a.

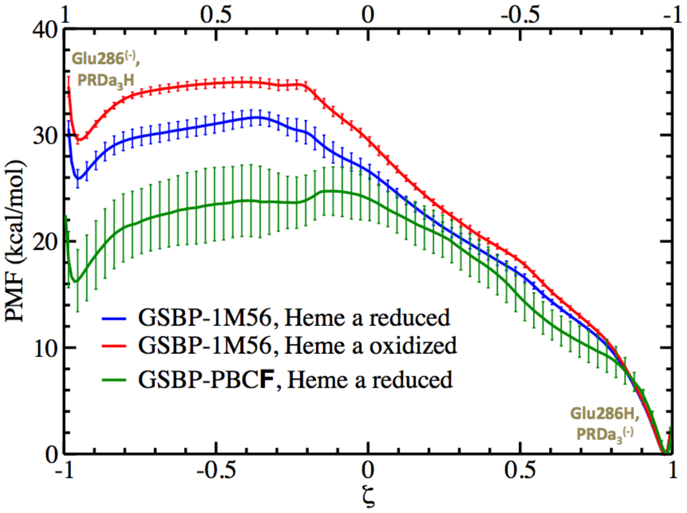


Figure S5: PMFs for direct proton transfer from Glu286H to $PRDa_3^{(-)}$ using different GSBP models and for different heme a oxidation states. These PMFs are calculated using the DFTB3-diag/MIO+gaus variant of SCC-DFTB. Compare to Figure 4 in the main text which show corresponding PMFs calculated by DFTB3/MIO/fit+gaus.

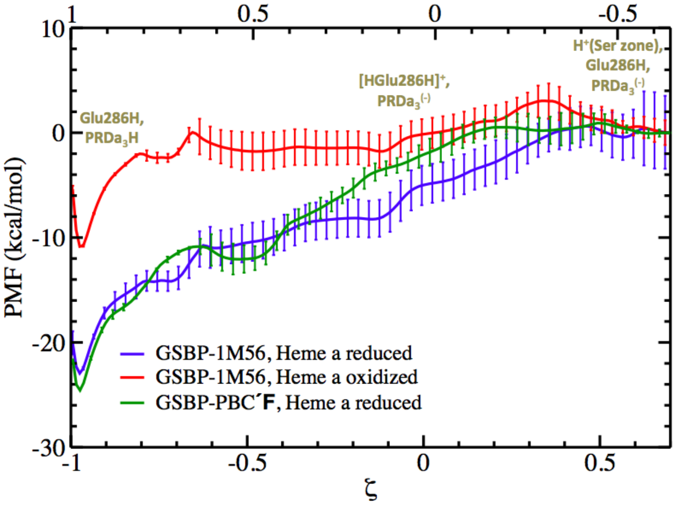


Figure S6: PMFs for 'concerted' proton transfer from Glu286H to $PRDa_3{}^{(-)}$ using different GSBP models and for different heme a oxidation states. These PMFs are calculated using the DFTB3-diag/MIO+gaus variant of SCC-DFTB. Compare to Figure 5 in the main text which shows corresponding PMFs calculated by DFTB3/MIO/fit+gaus. The much larger exothermicity for the PMFs here is due to the larger relative proton affinity errors in DFTB3/MIO/fit+gaus (7 kcal/mol) for protonated water clusters and carboxylic acids. The top X axis corresponds to the PMF for the ′**F** model while the lower X axis corresponds to the 1M56 PMFs.

As another way to gauge the computed proton transfer energetics, we compare the PMF results with thermodynamic integration (TI[5]) calculations for the $pK_a$ (or more rigorously, $pK_7'$, see discussions in Ref.[6]) of the proton donor/acceptor groups in the relevant proton transfer processes. Although sampling poses a major challenge in such QM/MM-TI based $pK_a$ calculations,[7–9] estimated $pK_a$ differences for the donor/acceptor groups should correlate with the proton transfer energetics from PMF calculations. All calculations discussed below use DFTB3-diag/MIO+gaus as the QM level in QM/MM-TI calculations; the computational details are similar to those reported in Ref.[6] and therefore not repeated here.

Considering first the direct proton transfer PMF in the 1M56 model with heme a reduced, we find that the protonation of $PRDa_3$ is ∼26 kcal/mol more unfavorable than the protonation of Glu286. The free energy of deprotonation $\Delta G^{(1)}_{E-RCOO(D/H)}$ of $PRDa_3$ in the 1M56, DXD-ROg model is ∼133.9 kcal/mol (from Table S4 below) while that for Glu286 in the 1M56, XDD-ROg model is ∼165.7 kcal/mol (from Table S6 in the **Supporting Infor-**

Table S4: Free energy derivatives[a], $\Delta G^{(1)}_{E-RCOO(D/H)}$ and statistical analysis of $pK_a$ simulations for various groups in CcO calculated using the DFTB3-diag/MIO variant of SCC-DFTB.

| | $PRDa_3$ $pK_a$ , DXD-ROg | | $PRDa_3$ $pK_a$ , PXD-ROg | $H_3O^+$ $pK_a$ , PDD-ROg | |
|---|---|---|---|---|---|
| $\lambda$ | GSBP-1M56 | GSBP-PBC′**F** | GSBP-1M56 | GSBP-1M56 | |
| | | | | "Serine zone" | Hydrophobic cavity |
| 0.00 | - | - | 194.6 (0.6) | 181.1(0.4) / 172.6(1.4) | 165.8 (0.5) |
| 0.20 | - | - | - | 150.3(1.5) / 155.0(0.3) | 149.7 (0.4) |
| 0.25 | 173.1 (0.5) | 182.2 (0.9) | 167.1 (0.9) | - | - |
| 0.40 | - | - | - | 115.5(0.8) / 116.9(0.3) | 129.2 (0.4) |
| 0.50 | 133.0 (0.3) | 143.1 (0.5) | 128.8 (0.7) | - | - |
| 0.60 | - | - | - | 95.3(0.8) / 96.8(0.5) | 107.4 (0.4) |
| 0.75 | 95.7 (1.0) | 112.2 (1.0) | 90.8 (0.8) | - | - |
| 0.80 | - | - | - | 63.1(2.1) / 57.9(1.3) | 81.8 (0.4) |
| 1.00 | 56.2 (0.9) | 51.5 (0.5) | 36.4 (3.6) | 27.4(1.1) / 43.6(0.1) | 42.1 (1.7) |
| $\Delta G^{(1)}_{E-RCOO(D/H)}$ [b] | 133.9(1.00) | 143.4 (0.98) | 123.5(0.99) | 105.4(1.00) / 107.1(0.99) | 112.6(0.98) |

| | $PRDa_3$ $pK_a$ , DXD-ROg | | | | $PRDa_3$ $pK_a$ , PXD-ROg | | $H_3O^+$ $pK_a$ , PDD-ROg | | | |
|---|---|---|---|---|---|---|---|---|---|---|
| | GSBP-1M56 | | GSBP-PBC′**F** | | GSBP-1M56 | | GSBP-1M56 | | | |
| | | | | | | | "Serine zone" | | Hydrophobic cavity | |
| $\lambda$ | prod(equ) | $\tau$(n) | prod(equ) | $\tau$(n) | prod(equ) | $\tau$(n) | prod(equ) | $\tau$(n) | prod(equ) | $\tau$(n) |
| 0.00 | - | - | - | - | 1.9(0.684) | 13(96) | 3.0(1.584) / 3.0(2.104) | 15(97) / 30(31) | 1.4(0.811) | 9(68) |
| 0.20 | - | - | - | - | - | - | 3.0(2.094) / 3.0(2.164) | 113(8) / 5(184) | 1.4(1.079) | 6(58) |
| 0.25 | 1.8(0.790) | 17(60) | 1.0(0.785) | 2(92) | 1.9(0.869) | 114(10) | - | - | - | - |
| 0.40 | - | - | - | - | - | - | 3.0(1.813) / 3.0(2.317) | 9(132) / 4(154) | 1.4(0.775) | 5(123) |
| 0.50 | 1.8(0.977) | 4(195) | 1.0(0.417) | 3(178) | 1.9(0.587) | 13(98) | - | - | - | - |
| 0.60 | - | - | - | - | - | - | 3.0(1.498) / 3.0(1.034) | 52(29) / 16(123) | 1.4(0.937) | 6(81) |
| 0.75 | 1.8(0.499) | 17(75) | 1.0(0.213) | 6(136) | 1.9(1.199) | 7(107) | - | - | - | - |
| 0.80 | - | - | - | - | - | - | 3.0 (2.232) / 3.0(0.571) | 23(34) / 105(24) | 1.4(0.635) | 13(61) |
| 1.00 | 1.8(0.994) | 22(36) | 1.0(0.218) | 3(230) | 1.9(1.491) | 68(7) | 3.0(1.094) / 3.0(0.935) | 13(142) / 15(135) | 1.4(0.651) | 66(12) |

[a] The free energy derivatives are given in kcal/mol, and the values in parentheses are statistical errors. [b] Computed on the basis of the linear fit of the free energy derivatives vs $\lambda$ and subsequent integration over $\lambda$; the values in parentheses are the $R^2$ values for the linear fit. [c] prod(equ) gives the total amount of simulation time (in nanoseconds) and the segment identified as equilibration (in parentheses). $\tau$ gives the size of the block (in picoseconds), and n gives the total number of blocks in the final free energy derivative calculations. The "reverse cumulative averaging" protocol of Yang et al.[7,10] was employed.

**mation** of Ref.[6]), making it more unfavorable to protonate $PRDa_3$ by 31.8 kcal/mol. $pK_a$ calculations are in general harder to converge compared to proton transfer PMF calculations, which may lead to the difference in the absolute numbers predicted by the two. However, the general trends agree very well between the two. For the **′F** model with heme a reduced, the direct proton transfer PMF predicts a protonated $PRDa_3$ to be less favored by ∼16 kcal/mol while $pK_a$ calculations predict this number to be ∼(158.1-143.4)=14.7 kcal/mol.

Table S4 below shows that $pK_a$ calculations predict that in the 1M56 model with heme a reduced, keeping Glu286 protonated and $PRDa_3$ deprotonated, moving a proton from the "Serine zone" in the D-channel to the hydrophobic cavity is more favorable by ∼(112.6-106.2)=6.4 kcal/mol. Again, keeping Glu286 protonated, moving the proton in the cavity to $PRDa_3{}^{(-)}$ is favored by ∼(123.5-112.6)=10.9 kcal/mol. Thus the overall drop in free energy when removing a proton from the D-channel to protonate $PRDa_3$ is ∼17.3 kcal/mol. The corresponding proton transfer PMF in Fig. S6 predicts a ∼12 kcal/mol drop in free energy from $\zeta$∼-0.5 (proton in cavity) to $\zeta$∼-1.0 (proton on $PRDa_3$) on the lower X-axis, which agrees well with the $pK_a$ predictions (10.9 kcal/mol). While the PMF predicts a much larger drop in the other parts (proton movement from D-channel to the cavity), the overall trends in the PMF that the proton is more stabilized as it moves from the D-channel to the hydrophobic cavity to $PRDa_3$ are well-matched by $pK_a$ predictions.

## Benchmarks for proton transfer involving a "doubly protonated" carboxylic acid

In order to benchmark the SCC-DFTB variants used to investigate the concerted proton transfer mechanism against higher level methods, a gas-phase model comprising of an acetic acid molecule, two water molecules and an excess proton is employed. For the SCC-DFTB variants, adiabatic maps along the $\zeta$ coordinate are obtained where $\zeta$=-1.0 and $\zeta$=1.0 correspond to proton localization on a water molecule while $\zeta$=0.0 roughly corresponds to a doubly protonated acetic acid (see Fig.S7 b,c). Single-point calculations are carried out

on DFTB3-diag/MIO+gaus structures (from the adiabatic map) with B3LYP/aug-cc-pVDZ and MP2/aug-cc-pVDZ. The energy profiles from the different methods (see Fig.S7 a) agree well with each other and predict that even in the gas-phase, a doubly protonated carboxylic acid is favored over a "hydronium". This is in agreement with the fact that the experimentally measured proton affinity of acetic acid is 187.3 kcal/mol[11] while that of water is 166.5±1 kcal/mol.[12]

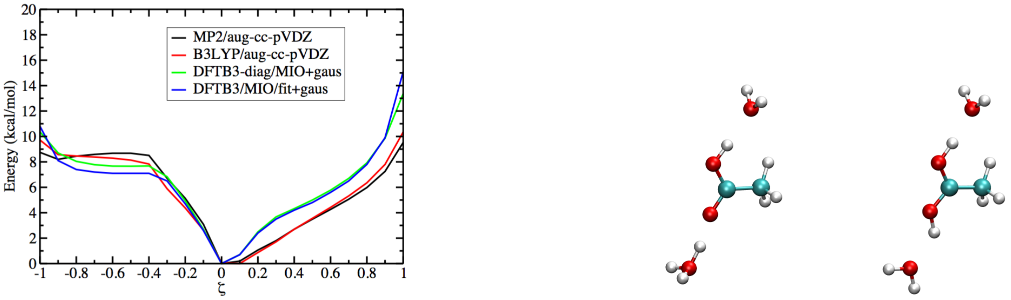


(a) (b) $\zeta$=-1.0 (c) $\zeta$=0.0

Figure S7: Benchmark for the doubly protonated glutamate using a gas phase model and different QM calculations. (a) Energy profile along $\zeta$ at different levels of theory. (b),(c) Configurations corresponding to $\zeta$=-1.0 and $\zeta$=0.0 respectively.

## Conformational isomers of the carboxylic group for Glu286

It is well known that the carboxylic group has two different isomers that feature different orientations of the acidic proton. We refer to them as the "*trans*" and "*cis*" isomers in this discussion (Fig. S8a). In the gas phase, the trans isomer is more stable and there is a significant barrier of about 10 kcal/mol for the isomerization between them. As shown in Table S5, both the barrier and energetic difference are well described by the DFTB3 variant used here and by the CHARMM force field, in comparison with *ab initio* calculations.

Table S5: Relative energy and isomerization barrier (all in kcal/mol) for the *trans* and *cis* isomers of acetic acid calculated by different methods.

| Method[a] | $\Delta E$ [b] | $\Delta E^{\ddagger}$ [c] |
|---|---|---|
| B3LYP/BSI | 5.3 | 12.8 |
| MP2/BSI | 5.3 | – |
| MP2/BSII//MP2/BSI | 5.2 | – |
| CHARMM22 | 6.8 | 12.9 |
| DFTB3 | 5.1 | 10.0 |

[a]Basis set I (BSI) is aug-cc-pVDZ; basis set II (BSII) is aug-cc-pVTZ. The DFTB3 variant used is DFTB3-diag/MIO. [b]The energy of the *cis* isomer relative to the *trans* isomer; see Fig.S8 for illustration. [c]The barrier is measured relative to the *trans* isomer.

In the context of CcO, the isomeric state of the carboxylic group might become relevant since with a *trans* isomer of Glu286H, the proton is not oriented properly to form a continuous water wire to either PRDa$_3$ or the BNC (e.g., see a snapshot in Fig. 3b in the main text). Therefore, we computed the PMF for the rotation of the acidic proton in Glu286H in the $\mathbf{P}_R$ state using pure MM simulations. As shown in Fig. S8b, the *trans* and *cis* isomers are energetically much closer in the enzyme than in vacuum. This is not difficult to rationalize; in the *cis* conformer, the carboxylic group forms better hydrogen bonding networks with the water molecules in the cavity.

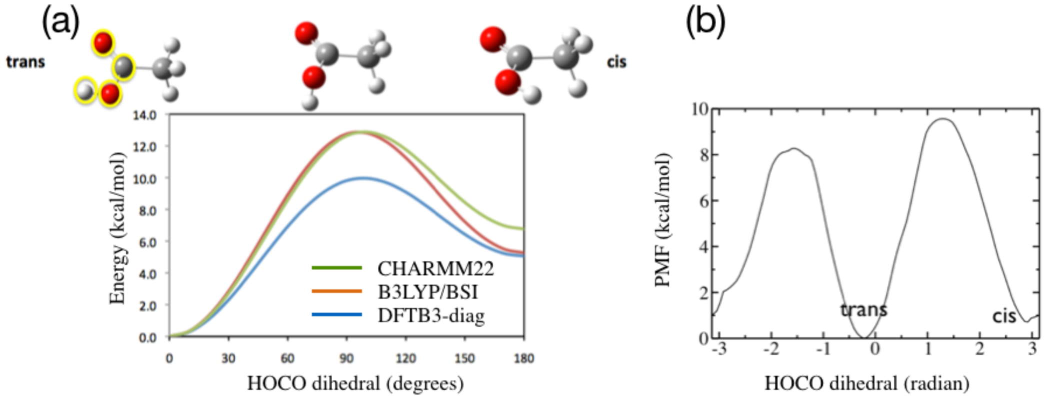


Figure S8: Isomerization of the carboxylic group in Glu286H between the "*trans*" and "*cis*" isomers. (a) Gas phase energies along the relevant dihedral angle computed at different levels; also see Table S5. (b) PMF along the same dihedral for Glu286H in the $\mathbf{P}_R$ state with pure MM (CHARMM 22) simulations.

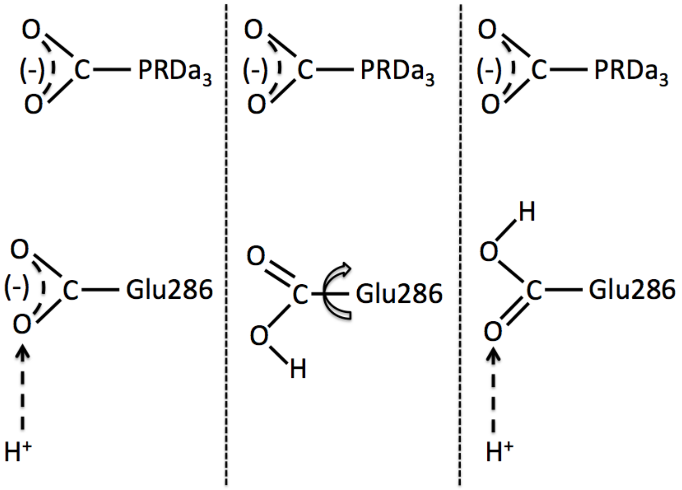


(a)

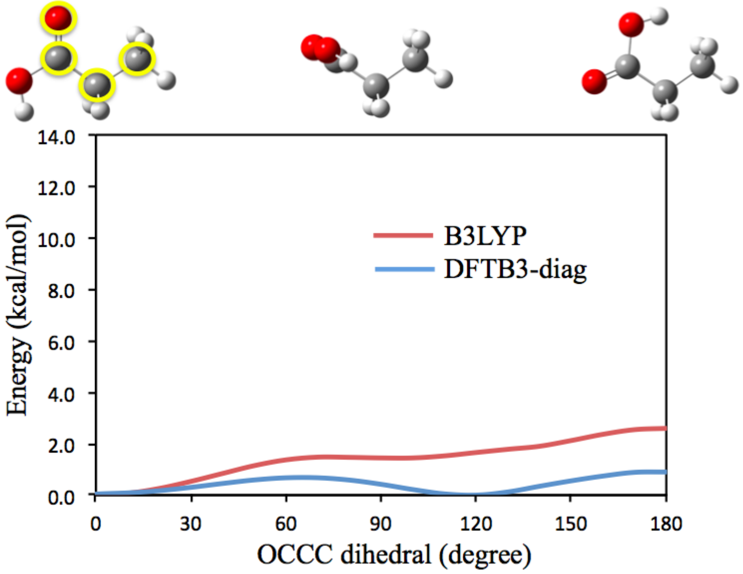


(b)

Figure S9: (a) Cis/trans isomerization of Glu286 does not need to be invoked, only rotation about a C-C single bond is required. (b) Potential energy profile for C-C rotation, in the same format as Fig.S8a; during the scan, the three C atoms are fixed to the coordinates in the optimized geometry for the cis conformation. The B3LYP calculations use the aug-cc-pVDZ basis set as in Fig.S8a.

Nevertheless, Fig. S8b indicates that there is a significant barrier of about 10 kcal/mol for the isomerization even in the enzyme. Does this contribute to the proton transfer kinetics? As shown schematically in Fig. S9, since Glu286 is expected to retain the "up" conformation[13] in the active site (protonation) states of physical importance, one does not have to

invoke any isomerization between the *cis* and *trans* isomers. Instead, the only isomerization required is a rotation around the C$\delta$ and C$\gamma$ bond, which is expected to be energetically lower compared to other processes studied; indeed, the intrinsic barrier for the corresponding C-C rotation in propionic acid is less than 3.0 kcal/mol at the B3LYP level.

# Additional simulation results

Figs. S10-S11 contain additional results related to the direct proton transfer from Glu286H to PRD$a_3$ (Fig. 4 in the main text); Figs. S12-S14 contain additional snapshots related to Figs. 6-8 in the main text.

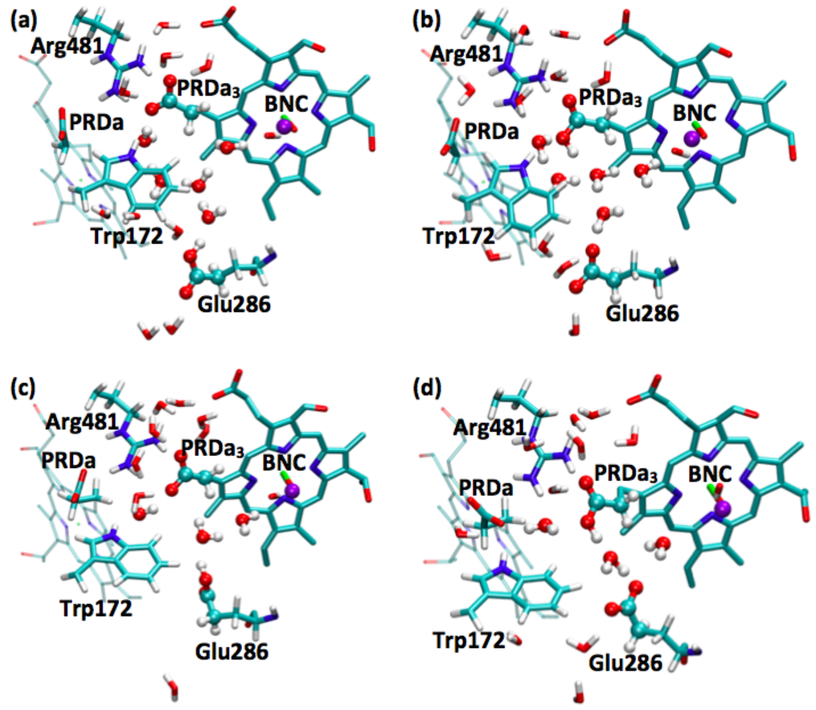


Figure S10: Representative snapshots from PMF calculations for the proton transfer from Glu286H to PRD$a_3$ (Fig. 4 of main text). (a) 1M56 simulations with Glu286H and PRDa$_3{}^{(-)}$; (b) 1M56 simulations with Glu286$^{(-)}$ and PRDa$_3$H; (c) pre$\mathbf{P}_R''$ simulations with Glu286H and PRDa$_3{}^{(-)}$; note the upward rotation of Glu286H (this corresponds to the shallow local minimum in the corresponding PMF in Fig. 4 of the main text); (d) pre$\mathbf{P}_R''$ simulations with Glu286$^{(-)}$ and PRDa$_3$H.

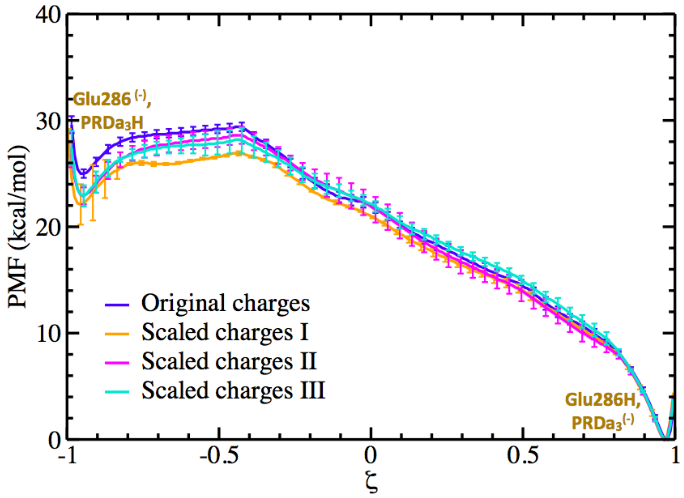


Figure S11: PMF for proton transfer from Glu286H to $PRDa_3^{(-)}$ in the 1M56 model with different charge-scaling schemes. 'Original charges' refers to unscaled charges for the MM atoms. 'Scaled charges I' involves scaling the charges of Arg481 by $1/\sqrt{2}$; 'Scaled charges II' involves scaling the charges of Arg481, Arg482, $PRAa_3$, PRDa and PRAa; 'Scaled charges III' involves scaling the charges of $Cu_B$ along with its ligands, in addition to scaling the charges of all groups under 'Scaled charges II'. Heme a is kept reduced in all these calculations.

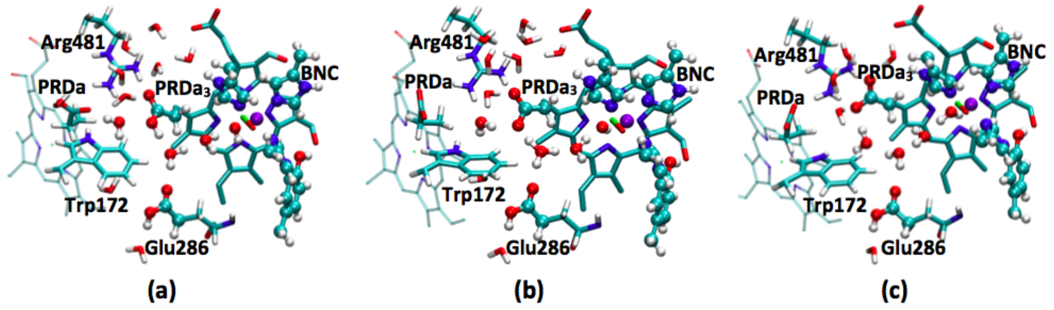


Figure S12: Snapshots from $PRDa_3H \rightarrow OH^{(-)}$-$Cu_B^{2+}$ proton transfer simulations (Figs. 6-7 in the main text) for the pre$\mathbf{P}''_R$ model depicting (a) a protonated $PRDa_3$ and a $OH^{(-)}$ bound to $Cu_B^{2+}$ (shown as a violet sphere); (b) proton in the cavity, coordinated directly to $PRDa_3^{(-)}$ ($\zeta$ ~-0.4 on the PMF); (c) $H_2O$ bound to $Cu_B^{2+}$ and $PRDa_3^{(-)}$ involved in a salt-bridge interaction with Arg481.

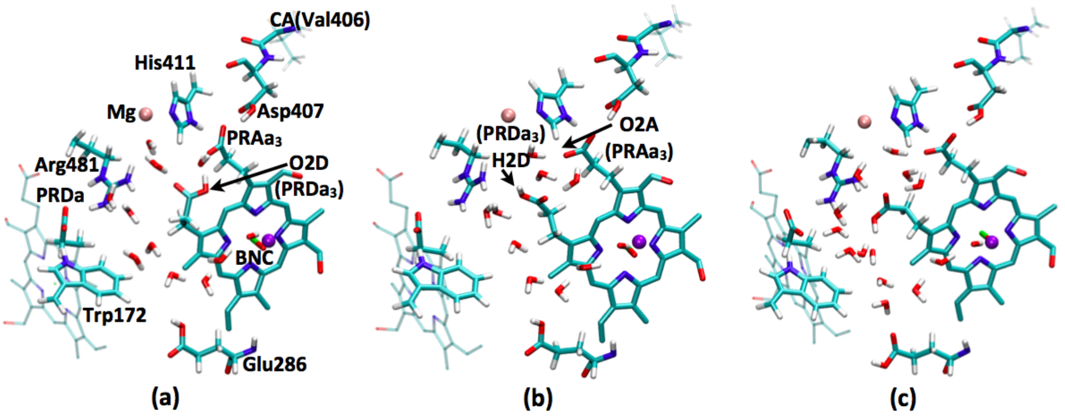


Figure S13: Representative snapshots from the MM PMF for PRDa$_3$H rotation in the $'\mathbf{F}$ model (Fig. 8 in the main text), after it has been loaded by a concerted mechanism: (a) Directly H-bonded configuration of PRDa$_3$H and PRAa$_3^{(-)}$. Also marked are the atoms CA (Val406) and O2D (PRDa$_3$), the distance between whom is chosen as the reaction coordinate for the PMF for PRDa$_3$H rotation. (b) PRDa$_3$H conformation corresponding to the shallow local minimum at 15 Å in the PRDa$_3$H rotation PMF. Also marked are the atoms H2D (PRDa$_3$) and O2A (PRAa$_3$), the distance between whom is chosen as the reaction coordinate for the QM/MM proton transfer PMF from PRDa$_3$H to PRAa$_3^{(-)}$. (c) PRDa$_3$H conformation corresponding to the minimum on the PRDa$_3$H rotation PMF at $\sim$16.8 Å. This shows PRDa$_3$H H-bonded to a cavity water molecule, after being loaded by a concerted mechanism (note the charge-neutral Glu286).

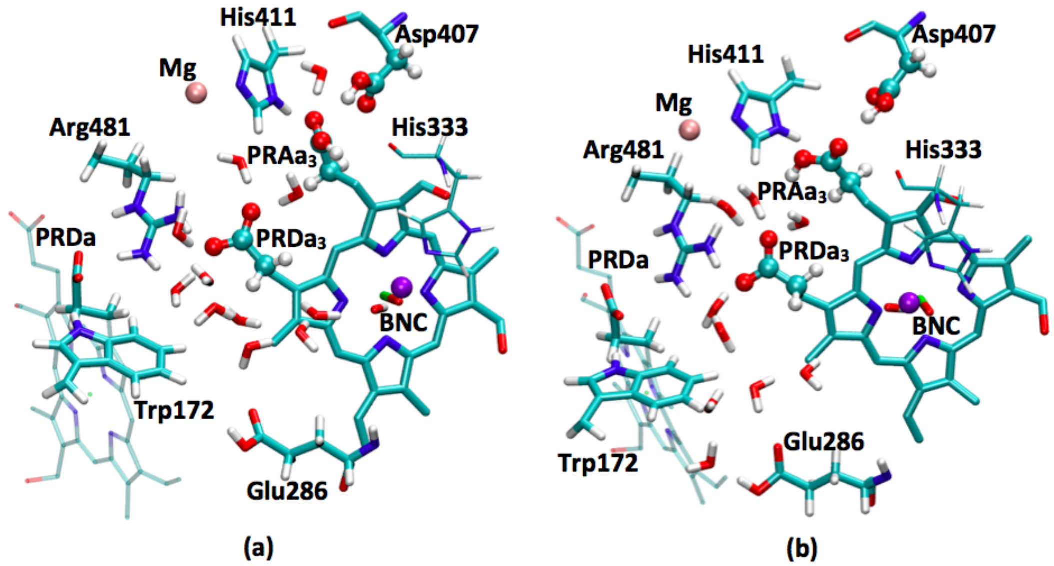


Figure S14: Snapshots from QM/MM PMF simulations for proton transfer from $PRDa_3H$ to $PRAa_3^{(-)}$ (Fig. 8 in the main text), showing a protonated $PRAa_3$ sampling a wide variety of conformations other than those in which it is directly H-bonded to $PRDa_3{}^{(-)}$. (a) $PRAa_3H$ weakly H-bonded to the backbone of His333, a $Cu_B$ ligand. (b) $PRAa_3H$ H-bonding to a water molecule.

## Proton uptake from Asp132 into the D-channel

The PMF and corresponding snapshots for the proton uptake from Asp132 to the serine zone in the D-channel are shown in Figs.S15-S16. As discussed in the main text, the calculations are done for an "in silico" double mutant, N139S/N121S. As the results illustrate, the bottleneck implicates proton transfers between water molecules hydrogen bonded to Ser139/Ser121. We note that Glu286 is ∼10 Å away from the Serine zone and more than 20 Å away from Asp132, and both regions are fairly polar in nature. Therefore, the protonation state of Glu286 is not expected to have a major impact on the proton uptake PMF. As shown in Fig.S17, this is indeed the case.

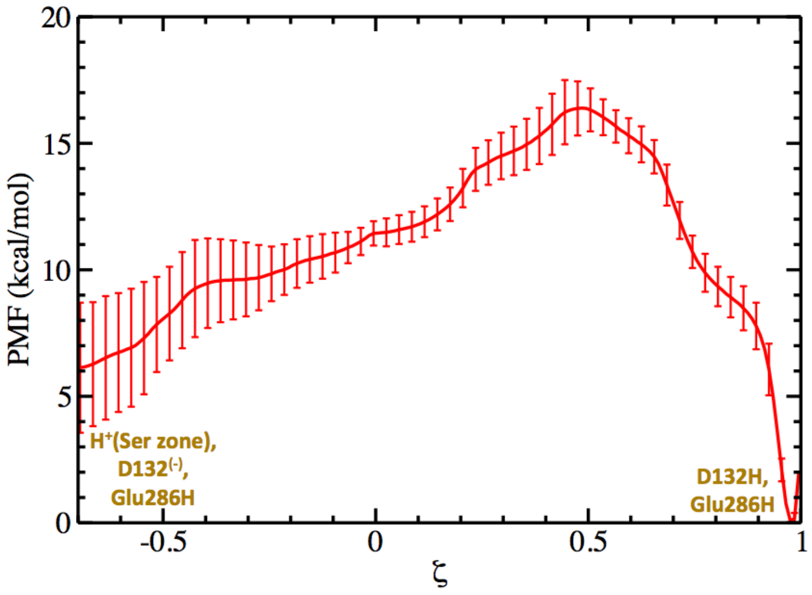


Figure S15: PMF for proton transfer from Asp132 located at the N-side mouth of the D-channel to the so-called “serine zone” in the D-channel in the N139S/N121S mutant.

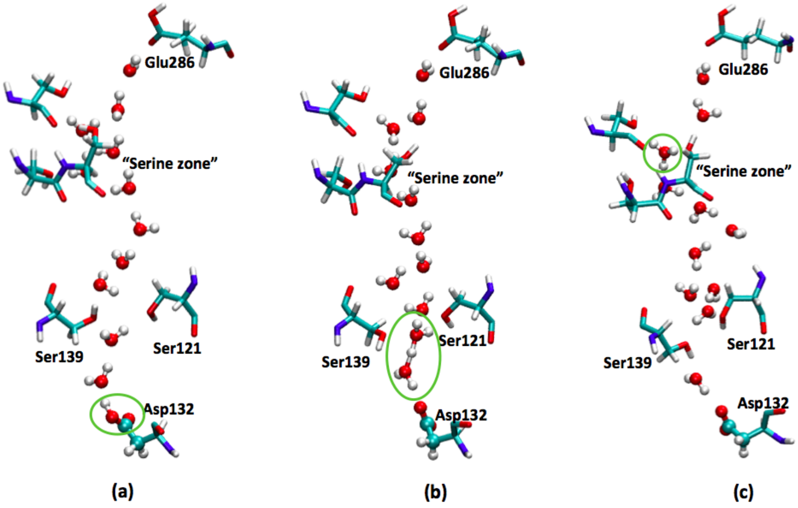


Figure S16: For proton transfer from Asp132 to the “serine zone” in the N139S/N121S mutant, snapshots representing (a) the “reactant” state with Asp132 protonated, this is the configuration from which proton transfer into the D-channel takes place. Asp132H, at equilibrium, samples a “downward” conformation, with its rotation being very facile;[14] (b) the “bottleneck” region on the PMF; (c) the “product” state with a hydrated proton in the “serine zone”.

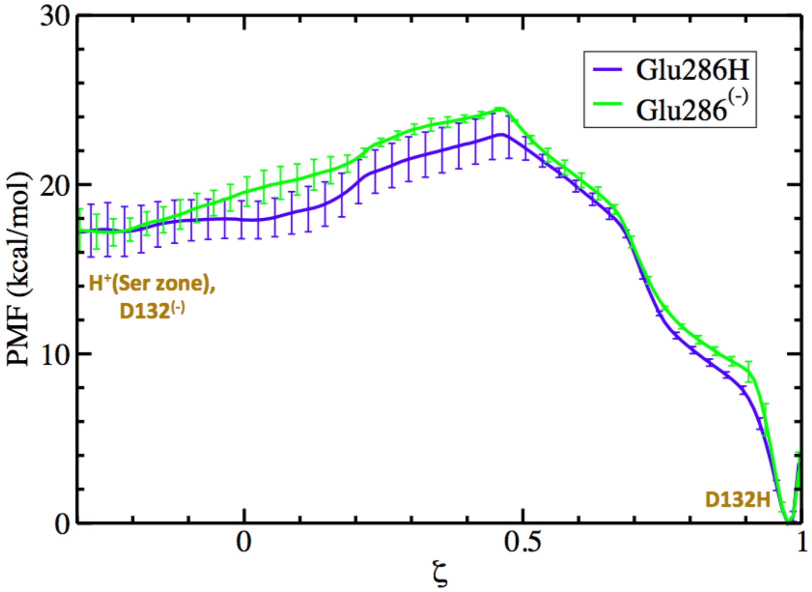


Figure S17: For different protonation states of Glu286, free energy profiles for proton transfer from Asp132 to the D-channel computed using DFTB3-diag/MIO+gaus. Glu286 protonation state is found to hardly have any effect on the profiles. The numerical difference between these PMFs and the one reported in Fig.S15 is consistent with the larger error for DFTB3-diag/MIO+gaus in the relative proton affinity of protonated water clusters and carboxylic acids.

# Additional Discussions

## Comparison to previous computational studies of proton transfers in CcO

As discussed in the main text, although there have been a significant number of computational studies of CcO, the analyses of proton transfers had various limitations. Among those, the work of Siegbahn and Blomberg focused on minimum energy path (MEP) calculations using active site models constructed based on crystal structures.[15–17] Although insightful ideas emerged from those calculations, the results of those MEP calculations without considering the thermal fluctuation of protein and internal water molecules are difficult to interpret; for CcO and other enzymes that catalyze proton transfers,[18,19] we found that MEP results differ greatly when different protein configurations are used: in carbonic anhydrase, for example,

depending on the initial configurations, local MEPs are either endothermic, thermoneutral or exothermic[18]. The studies of Voth and co-workers on CcO[20–23] has focused on proton transfers using fairly extensive MD sampling (though with protein backbone restrained to crystal positions) and the MS-EVB model, which has been extensively calibrated based on proton diffusion in bulk water.[24] However, in the published work so far, the deprotonation of Glu286 was not sampled explicitly; instead, a hydronium is placed next to a deprotonated/neutral Glu286.[23] Therefore, their results could not be used to compare the different proton transfer mechanisms as we did in this study. With a hydronium next to a neutral Glu286, the PMFs computed in Ref. 23 are also downhill in energetics, qualitatively similar to the results for the corresponding regions found here (Fig. 5 in the main text). A crucial difference is, however, the PMFs in Ref. 23 do not depend much on the oxidation state of heme a (i.e., compare OO and RO results in Fig. 7 of Ref. 23), while our results highlight the coupling between heme a reduction and PLS loading.

Warshel and co-workers have published extensive analyses of different aspects of CcO function,[25–30] including explicit considerations of the kinetics and thermodynamics of various proton transfer mechanisms. The semi-macroscopic analysis of Ref. 25,26 led to similar conclusion to the current work in that the proton transfer from Glu286H to $PRDa_3^{(-)}$ is energetically very unfavorable while the concerted proton transfer mechanism has more favorable barriers. In more recent work, however, they focused exclusively on the proton transfer from Glu286H to $PRDa_3^{(-)}$ and explored how the barrier changes when different numbers of water in the cavity and/or different side chain conformations are considered (though they acknowledged in Ref. 30 that the concerted proton transfer remains an important possibility to be explored using microscopic models). Specifically, they proposed that an "upward" rotation of Glu286 plays a major role in shortening the length of the water-mediated proton transfer pathway to $PRDa_3^{(-)}$, leading to a lower barrier of 12 kcal/mol and therefore preferential protonation of $PRDa_3^{(-)}$ over the BNC. By contrast, we find that the rotation of $PRDa_3^{(-)}$ in response to the presence of a proton in the cavity is what causes protonation of $PRDa_3^{(-)}$

prior to that of the BNC. Also, it is the presence of an *excess* proton coming from the D-channel that causes this "downward" rotation of $PRDa_3^{(-)}$. Without the excess proton, the "upward" rotation of Glu286H alone, which costs a notable amount of energy (~4 kcal/mol, see Fig. 4 in the main text), does not lead to the proton being thermodynamically favored to reside on $PRDa_3$.

Along this line, we note that both $PRDa_3$ and Glu286 are able to sample possible favorable orientations in the different GSBP models explored. In the 1M56 and 1M56+9w models, the cavity is "closed" with the Trp172 side-chain N hydrogen-bonded to $PRDa_3$, leading to a cavity free volume close to zero, thus limiting the flexibility of $PRDa_3$ and Glu286 side-chains. However, the pre$\mathbf{P}''_R$ model with a "partially open" and "dry" cavity is able to sample very "upward" and "downward" orientations of Glu286 and $PRDa_3$, respectively. In the $'\mathbf{F}$ model, the cavity is large and occupied by water molecules, with the proton transfer between Glu286 and $PRDa_3$ taking place via two mediating water molecules. The different PMFs (Fig.4 in the main text) indicate, once the energy penalty for the rotation of Glu286H is taken into consideration, the number of cavity water molecules that mediate proton transfer between Glu286H and $PRDa_3^{(-)}$ has only minor impact on the energetics; the major effect comes from the solvent stabilization of $Glu286^{(-)}$.

## Remaining unsettled questions: gate/valve for blocking proton back-flow and origin of decoupling mutations

Although the computational studies discussed so far help provide new ideas about the proton pumping cycle in CcO, several important mechanistic questions remain to be answered with compelling evidence and thus require further analysis with both experimental and computational studies.

First, the identity of the "gating element" that blocks proton back flow remains elusive. Previous MD simulation studies[31] have suggested that Glu286 functions as a valve. The essential observation was that with $PRDa_3^{(-)}$, the deprotonated Glu286 preferentially adopted

a "downward" orientation; thus it was suggested that after donating a proton to $PRDa_3$ (or a nearby group), the negatively charged Glu286 quickly isomerizes towards the "downward" orientation due to electrostatic repulsion with $PRDa_3^{(-)}$, cutting off potential back flow of proton. This trend was also seen in the more recent work of Knapp and co-workers;[32] similarly, in the MS-EVB simulations of Voth and Yamashita,[23] a deprotonated Glu286 quickly rotated to the downward orientation once the transferring proton passed through $PRDa_3$ toward $PRAa_3$.

Our previous MD simulations[13] with explicit membrane along with a careful consideration of protonation states of titratable residues found that with both $PRDa_3$ and Glu286 deprotonated, the Glu286 side chain has rather similar populations for the "upward" and "downward" orientations. However, we do not consider having both $PRDa_3$ and Glu286 deprotonated as a physically relevant enzyme state. Even if we assume that Glu286H is able to load $PRDa_3$ (which is *not* supported by our PMF calculations, Fig. 4 in the main text), we have a protonated $PRDa_3$ and deprotonated Glu286; with a concerted proton transfer mechanism, the state most vulnerable to proton back flow also has a loaded $PRDa_3$ and a deprotonated Glu286. Both our previous simulations[13] and the more recent explicit membrane MD simulation of Knapp and co-workers[32] found that with a loaded $PRDa_3$, a deprotonated Glu286 remained in the upward configuration for at least several nanoseconds since it is stabilized by water molecules in the cavity; we explicitly computed the free energy surface for the up/down isomerization of Glu286 and the result again indicated that the upward configuration is stable. It was suggested recently that since $PRDa_3$ might be only transiently protonated, one could study a state in which both $PRDa_3$ and Glu286 are deprotonated, and $PRAa_3$ protonated; in this state, Glu286 again preferred the downward configuration.[32] However, as explicitly shown here, there is no major driving force for the proton to transfer from $PRDa_3$ to $PRAa_3$ (Fig.8 in the main text). Therefore, even if Glu286 prefers a downward configuration with $PRDa_3^{(-)}$ and $PRAa_3H$, it is very easy for the proton to transfer back to $PRDa_3$, inducing the upward orientation of $Glu286^{(-)}$ and therefore back

flow of the proton.

In short, these considerations suggest that Glu286 isomerization *alone* is unlikely a robust gating element, a conclusion that we reached in Ref. 13 and also mostly supported by Knapp and co-workers in their recent study.[32] What would form a robust gate, however, remains unclear. The mechanism proposed here suggests that once the $PRDa_3$ is loaded with the concerted mechanism, the reverse barrier for the proton backflow from $PRDa_3H$ to the cavity/BNC is significant and in the 10 kcal/mol range (Figs. 5-6 in the main text). This large barrier can help $PRDa_3$ retain the proton during the nanosecond time-scale of rise in the cavity solvation level ($\mathbf{P}''_R$ PBC simulations show a high level of hydration in the cavity), post which, as is found by PMF calculations here, $PRDa_3H$ easily rotates "upwards" and donates the proton to $PRAa_3$ with small barriers of $\sim$2 kcal/mol (Fig. 8 in the main text). Although the thermodynamic driving force for the proton transfer to $PRAa_3$ is shallow ($\sim$ 2 kcal/mol), we note in simulations that a protonated $PRAa_3$ does not just remain H-bonded to $PRDa_3{}^{(-)}$ but samples a broad range of conformations (e.g., form H-bond to water molecules, Fig.S14). This provides the possibility that in the time-scale of proton transfer from Glu286H to the BNC in a $\mathbf{P}''_R$ state, the proton on $PRAa_3$ is transferred further to minimize the possibility of falling back to $PRDa_3{}^{(-)}$. Indeed, it is important that the proton be transferred away from $PRAa_3$ before BNC is protonated since calculations in the $'\mathbf{F}$ state show that a proton transfer from $PRDa_3H$ to $Glu286^{(-)}$ is highly favorable (similar to Fig.4 in the main text).

The missing piece of the puzzle is, however, following the same line of argument in the previous paragraph, that this constitutes a robust gating mechanism *only if* the proton is sufficiently stabilized in a region beyond $PRAa_3$ such that the barrier for back flow towards PRA/$PRDa_3$ is large. Therefore, it is important in the next step to examine proton transfers in regions beyond $PRAa_3$. This is an essential subject to study from another angle: since the concerted mechanism does not require Glu286H to deprotonate, what prevents the propionates of heme $a_3$ from accepting protons from the P-side of the membrane? The answer likely involves contributions from both electrostatic features and hydration patterns for the

protein regions that bridge heme $a_3$ and the lipid/protein interface near the P-side of the membrane. Indeed, it has been recognized that proton release from the loading site is a slow process (∼ms),[33] which indicates that significant barrier(s) separate the PLS region and the P-side.

Another important subject not clearly understood at all concerns the mechanistic origin of those "decoupling mutants" studied by various authors, most notably Gennis, Brzezinski and Michel.[34–40] Many such mutations occur at the entrance of the D-channel and are far (>20 Å away) from both Glu286 and the active site. In some of those mutants, the proton uptake kinetics are slowed down; thus one possibility is that delayed reprotonation of Glu286 would lead to enhanced chances for the proton on the PLS to fall back to either Glu286 or to the BNC, or for protons to enter from the P-side. In a more recent study,[41] however, the decoupling phenotype was also observed even when the proton uptake into the D-channel was found to increase. One common trend for the decoupling mutants was that the apparent $pK_a$ of Glu286 was always perturbed relative to the wild type enzyme. Therefore, one model that has been put forward[29] invoked that Glu286 samples two conformations (e.g., "up" and "down"), which have distinct intrinsic $pK_a$ values; the apparent $pK_a$ depends on the populations of the two conformations. It was suggested that mutations in the D-channel perturb the equilibrium between the two conformations by affecting the water structure in the D-channel, thereby altering the apparent $pK_a$ of Glu286; if only one of the conformations is able to deliver the proton to the PLS, then perturbation of this conformational equilibrium may lead to exclusive proton transfer to the BNC and abolish pumping. We note, however, both our work[6] and the study of Knapp et al.[32] did not find any strong dependence of Glu286 $pK_a$ on its side chain orientation. Clearly, a systematic analysis of the decoupling mutants with detailed microscopic models like those discussed here and kinetic network modeling is urgently needed.

## References


(1) Gaus, M.; Goyal, P.; Hou, G.; Lu, X.; Pang, X.; Zienau, J.; Xu, X.; Elstner, M.; Cui, Q. In *Molecular Modeling at the Atomic Scale: Methods and Applications in Quantitative Biology*; Zhou, R., Ed.; CRC Press, 2014; pp 33–82.

(2) Gaus, M.; Goez, A.; Elstner, M. *J. Chem. Theory Comput.* **2013**, *9*, 338–354.

(3) Yang, Y.; Yu, H.; York, D.; Cui, Q.; Elstner, M. *J. Phys. Chem. A* **2007**, *111*, 10861–10873.

(4) Gaus, M.; Cui, Q.; Elstner, M. *J. Chem. Theory Comput.* **2011**, *7*, 931–948.

(5) Li, G.; Cui, Q. *J. Phys. Chem. B* **2003**, *107*, 14521–14528.

(6) Goyal, P.; Lu, J.; Yang, S.; Gunner, M. R.; Cui, Q. *Proc. Natl. Acad. Sci. USA* **2013**, *110*, 18886–18891.

(7) Riccardi, D.; Schaefer, P.; Cui, Q. *J. Phys. Chem. B* **2005**, *109*, 17715–17733.

(8) Ghosh, N.; Cui, Q. *J. Phys. Chem. B* **2008**, *112*, 8387–8397.

(9) Riccardi, D.; Zhu, X.; Goyal, P.; Yang, S.; Hou, G.; Cui, Q. *Science China Chem.* **2012**, *55*, 3–18.

(10) Yang, W.; Bitetti-Putzer, R.; Karplus, M. *J. Chem. Phys.* **2004**, *120*, 2618–2628.

(11) Hunter, E. P. L.; Lias, S. G. *J. Phys. Chem. Ref. Data* **1998**, *27*, 413–656.

(12) Jursic, B. S. *J. Mol. Struct. (Theochem)* **1999**, *490*, 1–6.

(13) Yang, S.; Cui, Q. *Biophysical Journal* **2011**, *101*, 61–69.

(14) Henry, R. M.; Yu, C.-H.; Rodinger, T.; Pomes, R. *J. Mol. Biol.* **2009**, *387*, 1165–1185.

(15) Siegbahn, P. E. M.; Blomberg, M. R. A. *Biochim. Biophys. Acta* **2007**, *1767*, 1143–1156.

(16) Blomberg, M. R. A.; Siegbahn, P. E. M. *Biochim. Biophys. Acta* **2010**, *1797*, 129–142.

(17) Blomberg, M. R. A.; Siegbahn, P. E. M. *Biochim. Biophys. Acta* **2012**, *1817*, 495–505.

(18) Riccardi, D.; Schaefer, P.; Yang, Y.; Yu, H.; Ghosh, N.; Prat-Resina, X.; Konig, P.; Li, G.; Xu, D.; Guo, H.; Elstner, M.; Cui, Q. *J. Phys. Chem. B* **2006**, *110*, 6458–6469.

(19) Riccardi, D.; Yang, S.; Cui, Q. *Biochim. Biophys. Acta* **2010**, *1804*, 342–351.

(20) Xu, J.; Voth, G. A. *Proc. Natl. Acad. Sci. U.S.A.* **2005**, *102*, 6795–6800.

(21) Xu, J.; Sharpe, M. A.; Qin, L.; Ferguson-Miller, S.; Voth, G. A. *J. Am. Chem. Soc.* **2007**, *129*, 2910–2913.

(22) Lee, H. J.; Svahn, E.; Swanson, J. M. J.; Lepp, H.; Voth, G. A.; Brzezinski, P.; Gennis, R. B. *J. Am. Chem. Soc.* **2010**, *132*, 16225–16239.

(23) Yamashita, T.; Voth, G. A. *J. Am. Chem. Soc.* **2012**, *134*, 1147–1152.

(24) Swanson, J. M. J.; Maupin, C. M.; Chen, H.; Petersen, M. K.; Xu, J.; Wu, Y.; Voth, G. A. *J. Phys. Chem. B* **2007**, *111*, 4300–4314.

(25) Olsson, M. H. M.; Sharma, P. K.; Warshel, A. *FEBS Lett.* **2005**, *579*, 2026–2034.

(26) Olsson, M. H. M.; Warshel, A. *Proc. Natl. Acad. Sci. USA* **2006**, *103*, 6500.

(27) Pisliakov, A. V.; Sharma, P. K.; Chu, Z. T.; Haranczyk, M.; Warshel, A. *Proc. Nat. Acad. Sci. USA* **2008**, *105*, 7726–7731.

(28) Johansson, A.-L.; Chakrabarty, S.; Berthold, C. L.; Högbom, M.; Warshel, A.; Brzezinski, P. *Biochim. Biophys. Acta.* **2011**, *1807*, 1083–1094.

(29) Chakrabarty, S.; Namslauer, I.; Brzezinski, P.; Warshel, A. *Biochim. Biophys. Acta* **2011**, *1807*, 413–426.

(30) Chakrabarty, S.; Warshel, A. *Proteins: Struct., Funct., & Bioinf.* **2013**, *81*, 93–106.

(31) Kaila, V. R. I.; Verkhovsky, M. I.; Hummer, G.; Wikstrom, M. *Proc. Natl. Acad. Sci. U.S.A.* **2008**, *105*, 6255.

(32) Woelke, A. L.; Galstyan, G.; Galstyan, A.; Meyer, T.; Heberle, J.; Knapp, E. W. *J. Phys. Chem. B* **2013**, *117*, 12432–12441.

(33) Kaila, V. R.; Verkhovsky, M. V.; Wikström, M. *Chem. Rev.* **2010**, *110*, 7062–7081.

(34) Vakkasoglu, A. S.; Morgan, J. E.; Han, D.; Pawate, A. S.; Gennis, R. B. *FEBS Lett.* **2006**, *580*, 4613–4617.

(35) Han, D.; Namslauer, A.; Pawate, A.; Morgan, J. E.; Nagy, S.; Vakkasoglu, A. S.; Brzezinski, P.; Gennis, R. B. *Biochem.* **2006**, *45*, 14064–14074.

(36) Branden, G.; Pawate, A. S.; Gennis, R. B.; Brzezinski, P. *Proc. Natl. Acad. Sci. USA* **2006**, *103*, 317–322.

(37) Zhu, J.; Han, H.; Pawate, A.; Gennis, R. B. *Biochemistry* **2010**, *49*, 4476–4482.

(38) Lepp, H.; Salomonsson, L.; Zhu, J. P.; Gennis, R. B.; Brzezinski, P. *Biochim. Biophys. Acta* **2008**, *1777*, 897–903.

(39) Dürr, K. L.; Koepke, J.; Hellwig, P.; Müller, H.; Angerer, H.; Peng, G.; Olkhova, E.; Richter, O.-M. H.; Ludwig, B.; Michel, H. *J. Mol. Biol.* **2008**, *384*, 865–877.

(40) Brzezinski, P.; Johansson, A.-L. *Biochim. Biophys. Acta* **2010**, *1797*, 710–723.

(41) Johansson, A. L.; Hogbom, M.; Carlsson, J.; Gennis, R. B.; Brzezinski, P. *Proc. Natl. Acad. Sci. USA* **2013**, *110*, 8912–8917.
